# Synthesis and characterization of ethyl benzotriazolyl acrylate-based D–π–A fluorophores for live cell-based imaging applications†

**DOI:** 10.1039/c9ra00108e

**Published:** 2019-03-18

**Authors:** Ana Sofia Ortega-Villarreal, Eugenio Hernández-Fernández, Christopher Jensen, Gabriel A. Valdivia-Berroeta, Samuel Garrard, Israel López, Stacey J. Smith, Kenneth A. Christensen, Miguel A. Reyes-González, David J. Michaelis

**Affiliations:** Universidad Autónoma de Nuevo León, Facultad de Ciencias Químicas Pedro de Alba s/n, Ciudad Universitaria 66400 San Nicolás de los Garza Nuevo León Mexico eugenio.hernandezfr@uanl.edu.mx +52 81 1340 4890 ext. 6293 +52 81 1340 4890 ext. 6293; Department of Chemistry and Biochemistry, Brigham Young University 84602 Provo Utah USA dmichaelis@chem.byu.edu +1 801 422 9416; Universidad Autónoma de Nuevo León, UANL, Centro de Investigación en Biotecnología y Nanotecnología (CIBYN), Laboratorio de Nanociencias y Nanotecnología Autopista al Aeropuerto Internacional Mariano Escobedo Km. 10, Parque de Investigación e Innovación Tecnológica (PIIT) 66629 Apodaca Nuevo León Mexico

## Abstract

A series of eight new ethyl (*Z*)-benzotriazolyl acrylates 6a–d and 7a–d have been synthesized by conventional heating and microwave irradiation from ethyl benzotriazolyl acetates 3 and 4 with the corresponding aromatic aldehydes. This work reports the synthetic approach and spectroscopic characterization (^1^H, ^13^C-NMR, HRMS) of all the synthesized compounds. X-ray diffraction analyses were performed for molecules 6a, 7a and 7d. Photophysical properties of compounds were evaluated. Finally, compound 6a was tested in a human cell line and showed low to no cytotoxicity at relevant concentrations. Initial testing demonstrates its potential use as a fluid-phase fluorescent marker for live cell imaging.

## Abstract 2

Synthesis of eight new benzotriazolyl derivatives, characterization, X-ray diffraction analyses, photophysical properties and their application as safe-fluorophores in mammalian cells.

## Graphical abstract

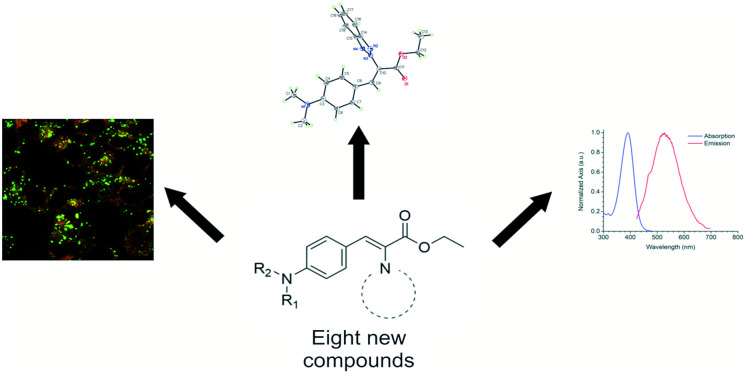

## Introduction

Organic fluorescent molecules have gained great attention in recent decades due to their diverse applications both in chemistry [organic light-emitting diodes (OLED),^1–3^ organic photovoltaic devices (OPV),^4,5^ organic field-effect transistors (OFET),^6,7^ organic thin film transistors (OTFT), metal ion sensors^8^] and in biology (fluorescent tags,^9,10^ pH monitoring in biological systems,^11^ biological species sensors^12,13^ and cell bioimaging^9,14–16^). The possibility of using this class of fluorescent compounds for these applications is related to their chemical (reactivity, solubility, lipophilicity and stability) and photophysical properties [excitation maximum (*λ*_ex_), emission maximum (*λ*_em_), extinction coefficient (*ε*), quantum yield (*ϕ*), lifetime of the excited state and photostability].^17,18^

The design and synthesis of fluorescent molecules requires incorporating certain structural characteristics that include multiple combined aromatic groups, planar or cyclic molecules with several π bonds, conjugated double bonds, and resonance.^19^ α,β-Unsaturated carbonyl compounds and benzotriazoles are examples of structural motifs that could be promising fluorophores and which are of particular interest in our research groups. Benzotriazole derivatives are well-known for their biological applications,^20–22^ including their use as antibacterial,^23^ antitubercular,^24^ antifungal,^25^ antiviral,^26^ antiinflammatory,^27^ anticonvulsant,^28^ and anticancer^29^ agents. In addition, the extended conjugated system in benzotriazoles makes them promising candidates for biological probes that exhibit fluorescence.^30,31^ Thus, our goal from the outset was to explore how the biocompatible structure of the benzotriazole could be combined with their fluorescent properties to construct fluorescence probes capable of measuring a variety of biological processes.

Preparation of α,β-unsaturated carbonyls can be achieved through several conventional routes including aldol condensation, carbon halogenation followed by elimination, oxidation of allylic alcohols and Wittig reactions, among others.^32^ An alternative route to synthesize these compounds is the Doebner modification^33^ of the Knoevenagel condensation,^34^ which can be carried out in the presence of carboxylic acid functional groups. Nevertheless, preparation of α,β-unsaturated esters incorporating a benzotriazole group in the α position has not been reported and this approach could afford a practical and useful series of new compounds with enhanced fluorescent properties. Herein, we report the synthesis of eight ethyl benzotriazolyl acrylates using conventional heating and microwave irradiation techniques. Additionally, the crystallographic and photophysical properties of these new compounds are reported. Cytotoxicity, cell labeling and localization of one of the compounds are tested.

## Results and discussion

### Synthesis of ethyl benzotriazolyl acrylates 6a–d and 7a–d

**Scheme 1:**
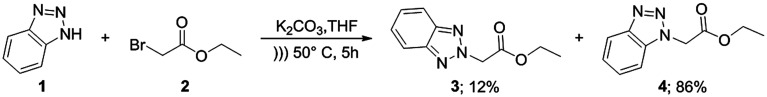
Synthesis of ethyl benzotriazolyl acetates 3 and 4.

For the synthesis of target compounds 6a–d and 7a–d, benzotriazole was reacted with ethyl bromoacetate and K_2_CO_3_ as a base to obtain precursors 3 and 4 (Scheme 1). In this reaction, both the 1*H* and 2*H*-isomers of benzotriazole were obtained due to the inherent tautomeric structure of benzotriazole.^20,35^ In our synthesis, the 1*H*-isomer was obtained in greater yield (86% compared to 12% yield). These results agree with chemical evidence supported by ultraviolet, infrared and proton nuclear magnetic resonance spectra studies indicating the predominance of the 1*H*-isomer at room temperature.^36–38^ In addition, the large dipole moment of the 1*H*-tautomer enhances its stability over the 2*H*-isomer because of the different intermolecular interactions taking place either with itself (in the solid state) or with the solvent (in solution).^35^

Compounds 3 and 4 served as common intermediates for the preparation of all our acrylate derivatives *via* condensation with commercially available aromatic aldehydes. The α,β-unsaturated acrylates 6a–d and 7a–d were synthesized by Knoevenagel condensation reaction between 3 and 4 and a variety of aromatic aldehydes, including 4-(dimethylamino)benzaldehyde, 4-(diethylamino)benzaldehyde, 4-(diphenyl-amino)benzaldehyde and 4-(4-morpholinyl)benzaldehyde (5a–d). This condensation reaction was conducted using piperidine as the stoichiometric base and ethanol as solvent (Table 1).

**Table 1:** Synthesis of ethyl benzotriazolyl acrylates 6a–d and 7a–d

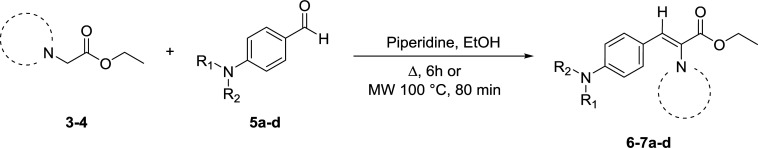
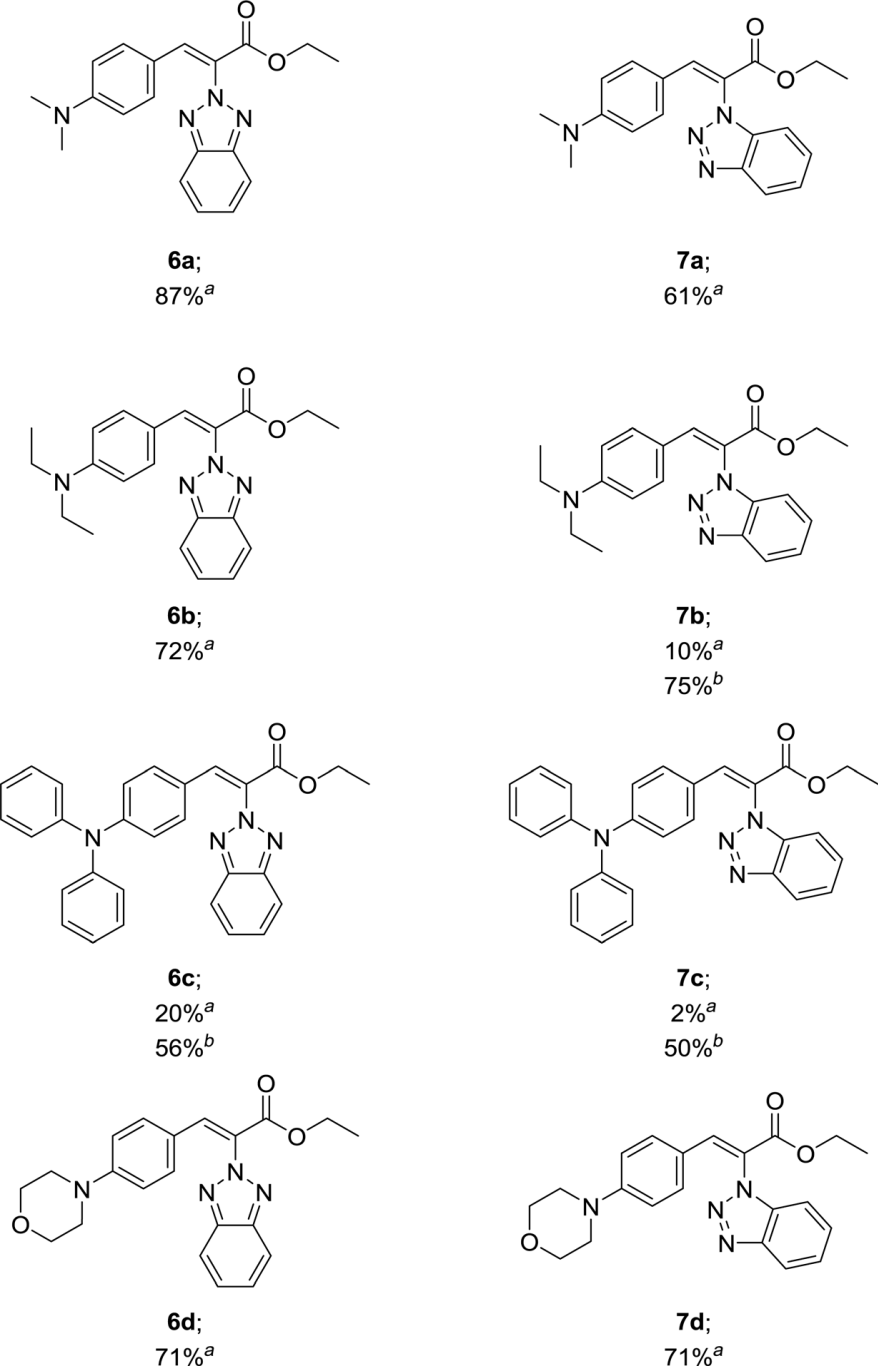

a Conventional heating.

b Microwave irradiation.

Our results in Table 1 demonstrate that the reaction of compound 3 with aromatic aldehydes 5a–d afforded ethyl benzotriazolyl acrylates 6a–d with yields ranging from 20% to 87% under reflux conditions. Good yields were obtained for compounds 6a, 6b and 6d. However, compound 6c was obtained with a 20% yield. We suspected that the low yield could be strongly influenced by the lower reactivity of the 4-(diphenylamino)benzaldehyde; therefore, we decided to carry out the reaction under microwave irradiation, in accordance with our previous studies.^39,40^ We found that at 100 °C for 80 min, the yield of 6c increased from 20% to 56%. The reaction of benzotriazole 4 with aromatic aldehydes 5a and 5d afforded ethyl benzotriazolyl acrylates 7a and 7d with yields of 61% and 71%. In a similar way, we performed the microwave irradiation reaction for compounds 7b and 7c and we were able to increase the yields from 10% and 2% up to 75% and 50%, respectively.

### Single-crystal X-ray diffraction analyses

**Fig. 1:**
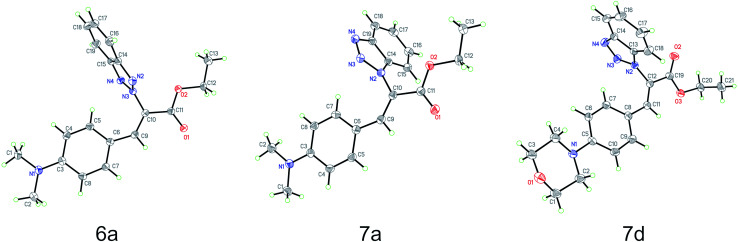
Molecule structures of ethyl benzotriazolyl acrylates 6a (CCDC 1882468), 7a (CCDC 1867840) and 7d (CCDC 1882467) determined by single-crystal X-ray diffraction analysis.

Based on the crystal structures of 6a, 7a and 7d, the (*Z*)-geometry of the double-bond was unequivocally confirmed (Fig. 1). The angle between the benzene and benzotriazole rings in each structure was calculated using the non-hydrogen atoms to define planes containing each ring; the angles between these planes were found to be 87.7°, 72.6° and 76.9°, respectively. This near perpendicular orientation is likely due to steric hindrance between the aromatic rings. Details of the crystal structure of 6a, 7a and 7d derivatives are listed in Table 2.

**Table 2:** Crystallographic data and refinement metrics for 6a, 7a and 7d

Compound	6a	7a	7d
Chemical formula	C_19_H_20_N_4_O_2_	C_19_H_20_N_4_O_2_	C_21_H_22_N_4_O_3_
*M*	336.39 g mol^−1^	336.39 g mol^−1^	378.42 g mol^−1^
Crystal system	Monoclinic	Monoclinic	Orthorhombic
Space group	*P*2_1_/*c*	*P*2_1_/*c*	*Pca*2_1_
*a*	9.0183(7) Å	13.2422(7) Å	17.5573(10) Å
*b*	8.3790(7) Å	8.7080(5) Å	6.3237(4) Å
*c*	23.0425(18) Å	29.4352(16) Å	33.7271(19) Å
*α*	90°	90°	90°
*β*	97.233(4)°	96.420(3)°	90°
*γ*	90°	90°	90°
*V*	1727.3(2) Å^3^	3373.0(3) Å^3^	3744.6(4) Å^3^
*R*_1_	0.0366	0.0369	0.0362
w*R*_2_	0.0943	0.0944	0.0958

### Photophysical properties

**Fig. 2:**
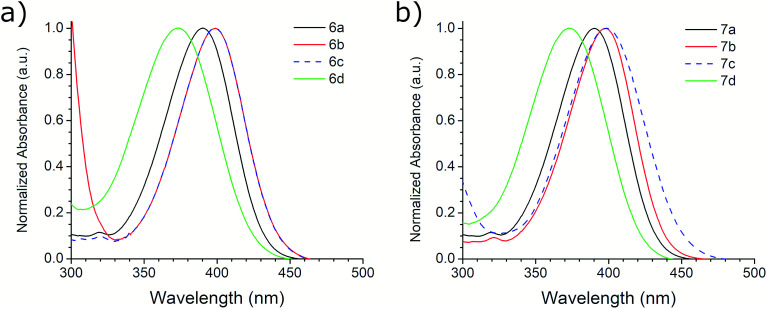
Absorption spectra of (a) 6a–d and (b) 7a–d ethyl benzotriazolyl acrylate derivatives.

The photophysical properties of compounds 6a–d and 7a–d were analyzed in solution, at 10^−5^ M, using methanol as solvent. Fig. 2 shows the absorption bands of these compounds, which are located between 300 and 450 nm, and are attributed to n–π* transitions; absorbance data were normalized. The wavelengths of maximum absorption, *λ*_Abs_, for each derivative in both symmetrical and asymmetrical isomers are the same. Compounds 6b-c and 7b-c presented an equal value of *λ*_Abs_, and compounds 6b and 6c also showed the same absorption pattern between 340 and 460 nm. However, 6b uniquely presents a second absorption band at 271 nm, which could be attributed to a second chromophore, but we did not record an emission spectrum due to its short value (<400 nm) (Fig. S20, ESI†). Table 3 shows the *λ*_Abs_ values for each compound and their corresponding photon energy, *E*_Abs_. Similar to the behavior of *λ*_Abs_, the extinction coefficient, *ε*, for each derivative in both isomeric series are of comparable magnitude and presented high values (>30 000 M^−1^ cm^−1^), making them suitable for biological and medical applications.^41^

**Table 3:** Photophysical properties of 6 and 7 derivatives in methanol

Compound	*λ*_Abs_ (nm)	*E*_Abs_ (eV)	*ε* (10^4^ M^−1^ cm^−1^)	*λ*_Em_ (*λ*_Ex_) (nm)	*E*_em_ (eV)	SS (nm)	SS (eV)
6a	391	3.17	3.28	519 (416)	2.39	128	0.78
6b	399	3.11	3.32	463 (363)	2.68	64	0.43
6c	398	3.12	4.26	463 (363)	2.68	65	0.44
6d	372	3.33	2.79	505 (340)	2.46	133	0.88
7a	390	3.18	3.27	467 (413)	2.66	77	0.52
7b	398	3.12	3.37	466 (398)	2.66	68	0.45
7c	398	3.12	4.26	566 (395)	2.19	168	0.92
7d	372	3.33	2.79	467 (393)	2.66	95	0.68

**Fig. 3:**
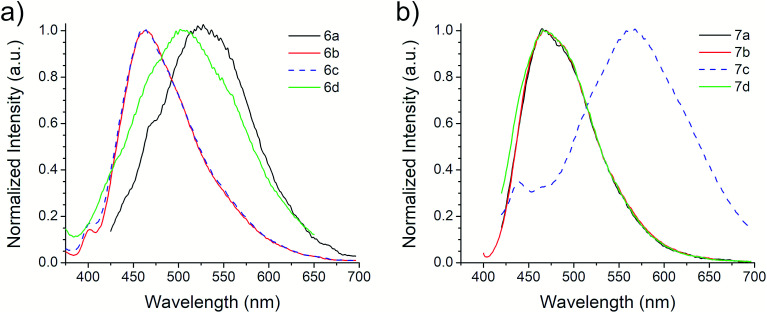
Emission spectra of (a) 6a–d and (b) 7a–d ethyl benzotriazolyl acrylate derivatives.

The first excitation wavelength probed for each compound was *λ*_Abs_, then the wavelength of maximum emission, *λ*_Em_, was fixed and the excitation spectra were collected. After that, the maximum of the excitation spectra, *λ*_Ex_, was fixed as corrected excitation wavelength, only in the case of 6b derivative, the *λ*_Em_ was shifted after this correction. The emission spectra collected with the corrected excitation wavelength are shown in Fig. 3. The emission pattern of 6b and 6c derivatives is the same, which was expected due to observations in the UV-vis spectra. The UV spectra indicates that the excitation and emission processes for both molecules possess the same energy levels. Therefore, we can conclude that their non-radiative processes are equivalent.^42^

The Stokes shift, SS, is the difference between the absorption and emission maxima,^43^ and is indicated in the following equation:

1 SS = *λ*_Em_ − *λ*_Abs_

A large SS is one important property that should be considered when identifying potentially useful fluorescent markers or labels for cell-based analysis. Table 3 shows the SS values in nm for derivatives 6 and 7, and their equivalence in eV. In contrast with the other photophysical parameters previously mentioned, the SS value does not appear to follow a trend that depends on the substituent on the aromatic aldehyde or on the substitution of the benzotriazole ring. From these studies, we selected derivative 6a for further photophysical and biological testing due to the combination of its features: high chemical yield, visible *λ*_Abs_, high *ε*, and large SS.

**Fig. 4:**
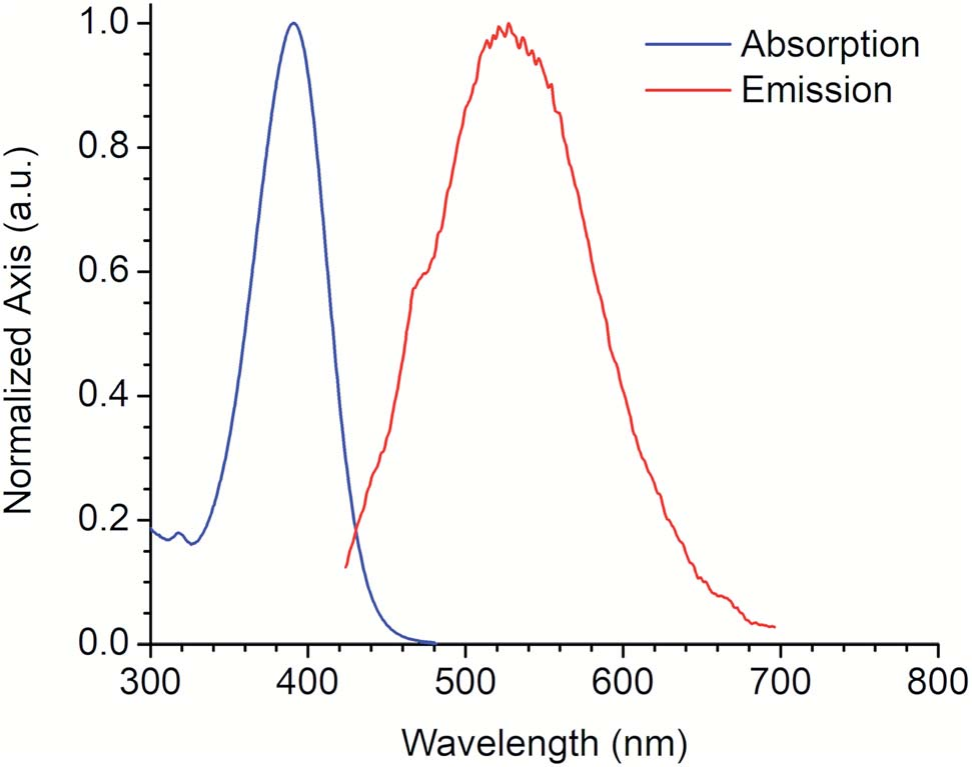
Absorption and emission spectra of 6a derivative.

Fig. 4 shows the absorption and emission spectra of the 6a derivative depicting its large SS (128 nm) like other commonly used fluorophores with similar properties (*e.g.* Lucifer yellow, Pacific orange) that are used in fluorescence microscopy and flow cytometry. Compound 6a was tested as fluorescent label for mammalian cells. An additional experiment using a DMSO stock solution (needed for cell-based assays) was performed in order to confirm that its photophysical properties were not significantly modified (Fig. S21, ESI†).

### *In vitro* cytotoxicity and cell imaging

**Fig. 5:**
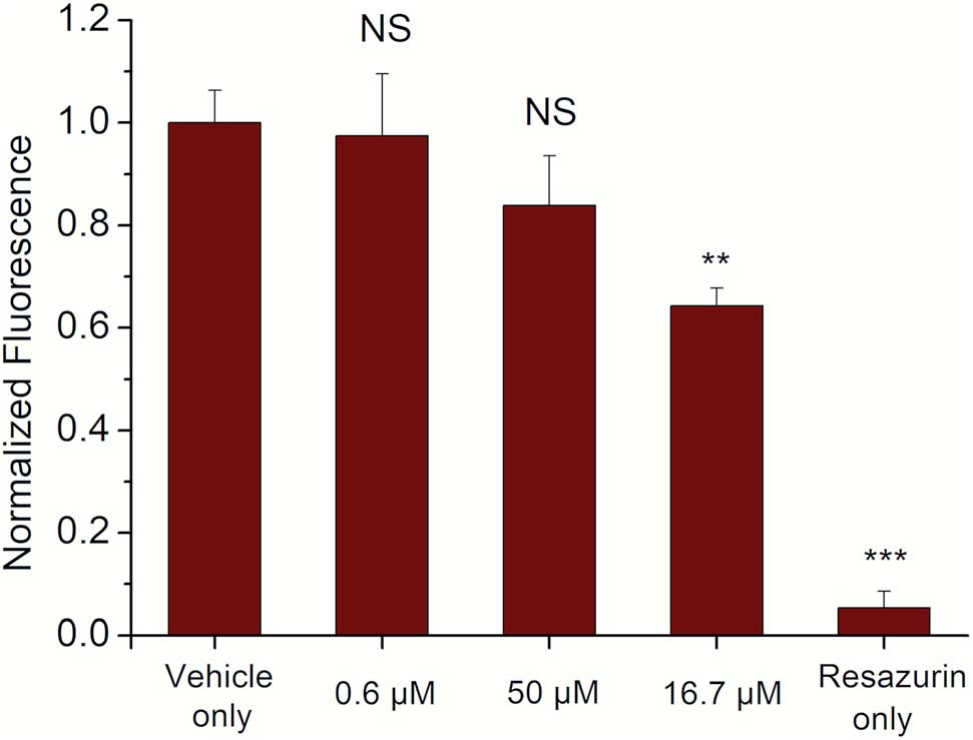
Cell toxicity of compound 6a at various concentrations. After 24 h incubation with derivative 6a at 50 μM, cells retained 64 ± 4% viability compared to control cells. Incubation with 0.6 μM 6a appeared to have negligible cytotoxicity. Significance was assessed relative to positive control using a two-tailed *t* test (NS: no significance, **P < 0.01, ***P < 0.001).

The cytotoxicity of the selected derivative 6a was assessed following a 24 h treatment to the cells by measuring conversion of resazurin to resorufin, a common indicator of cell viability, over a 4 h period. The results showed limited cytotoxicity of compound 6a for HEK 293T cells (a human cell line) even at concentrations of 50 μM that are about 5× greater than would typically be used. At this concentration, cell viability was reduced to only 64 ± 4% compared to the vehicle control (Fig. 5).

Based on this data, compound 6a appears to be similarly or less toxic than other commonly-used fluorophores such as Rhodamine 6G and Cy5.5 indicating its potential for use as a marker for *in vitro* and *ex vivo* cell studies.^44^ Additionally, compound 6a exhibits a unique absorption–emission profile compared to many other mammalian cell-safe fluorophores,^45^ which may further promote its utility in these types of assays.

**Fig. 6:**
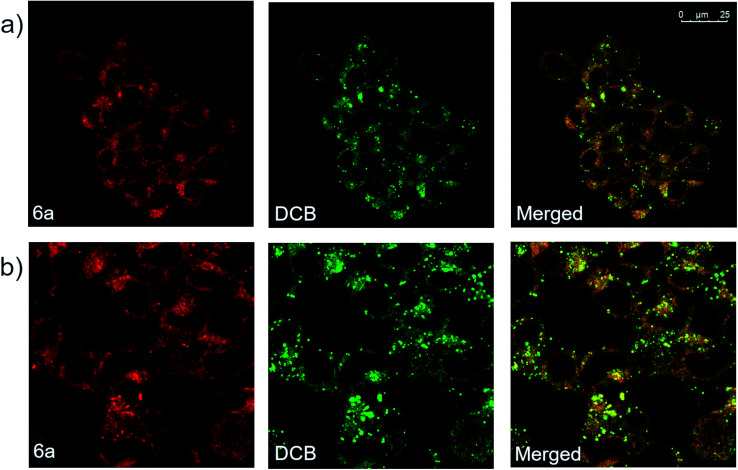
(a) Confocal fluorescence microscopic images of HEK 293T cells incubated with derivative 6a (10 μM) and DCB (50 μg mL^−1^) for 16 h, and (b) confocal fluorescence microscopic images of modified assay in which HEK 293T cells were incubated with 6a (10 μM) and DCB (50 μM) for 20 h, then 6a (10 μM) alone for 4 h.

Derivative 6a was also analyzed *via* confocal microscopy for cellular staining, uptake and localization in HEK 293T cells. Many fluorescent molecules and nanoparticles are largely internalized by cells *via* endocytosis. To determine localization of 6a, cells were co-incubated with 50 μg mL^−1^ of Dextran–Cascade Blue, DCB, a fluorescent conjugate which localizes to endosomes^46^ and eventually fuses with lysosomes.^47^ Confocal analysis of cells revealed that derivative 6a accumulated in vesicle-like structures located in the cytosol, often co-localizing with DCB (Fig. 6a). This suggests that 6a is localized to endosomes and/or lysosomes when taken up by the cell. Additionally, a modified experiment performed by removing DCB after 4 h prior to imaging showed broad co-localization of the two fluorophores once again, indicating that 6a is indeed trafficked along the endocytic pathway to late endosomes and lysosomes (Fig. 6b). Based on these results, it appears that the cellular localization and fates of 6a and dextrans are similar making 6a a fluid-phase probe similar to conjugated dextrans. It is anticipated modifications could be made to the compound to change the mechanism of uptake and localization in cells without disrupting the fluorophore. In addition, small molecular weight fluorescent compounds, like those described here, are uniquely suited as minimally perturbing labels for larger biomolecules and even metabolites for use in cell-based assays and imaging.

## Conclusions

In this work, we present eight ethyl benzotriazolyl acrylate derivatives prepared using the Knoevenagel condensation reaction with yields from 50% to 87%. In general, the synthesis was efficient using conventional heating methods, yet the yields improved when compounds 6c, 7b and 7c were synthesized under microwave irradiation conditions. The (*Z*)-geometry of double-bond was confirmed for compounds 6a, 7a and 7d by single-crystal X-ray diffraction. The optical properties assay showed that compounds 6a, 6d and 7c presented a wider Stoke shift, therefore, derivative 6a was employed to evaluate its toxicity and to generate bioimages. Finally, based on the relatively mild toxicity, broad Stokes shift, and definitive cellular localization data collected for 6a, we have demonstrated the potential of this kind of molecules to serve as a novel cell-safe fluorophore that can be used in a variety of assays.

## Experimental

### Materials and instruments

All commercial materials were used as received unless noted otherwise. Melting points were registered using an Electrothermal Mel-Temp apparatus and are uncorrected. Thin-layer chromatography was performed on pre-coated sheets of silica gel 60 F254 (E. Merck). For column chromatography 230–400 mesh silica gel 60 (E. Merck) was used as stationary phase. The spectra were obtained in CDCl_3_ solution using TMS as the internal reference. ^1^H NMR data were acquired on an Inova 300 MHz, an Inova 500 MHz, or an NMR-S 500 MHz spectrometer. Chemical shifts (*δ*) are reported in parts per million and coupling constants were expressed as (*J*) and reported in Hertz (Hz). Multiplicities are reported as follows: s (singlet), d (doublet), t (triplet), q (quartet), m (multiplet). ^13^C NMR data were acquired on an Inova at 75 MHz or Inova NMR-S spectrometer at 125 MHz. Mass spectral data were obtained using ESI techniques (Agilent, 6210 TOF). Reactions carried out with stirring under microwave irradiation in closed-vessels were all performed with a CEM Discover Synthesizer. For the photophysical characterization reagent-grade methanol was used as solvent. UV-vis absorption spectra were recorded on a Shimadzu 1800 spectrophotometer using quartz cuvettes (1 cm path length). Emission spectra were measured on a Perkin-Elmer LS 55 fluorescence spectrophotometer using quartz cuvettes (1 cm path length). For *in vitro* cytotoxicity and confocal microscopy assays, HEK 293T cells (ATCC) were cultured at 37 °C and 5% CO_2_ in Dulbecco's Modified Eagle Medium (GenClone) supplemented with 10% fetal bovine serum (VWR). For cell-based assays, cells were trypsinized with 0.25% trypsin solution in HBSS (GenClone) and seeded in 96-well optical plate (Nunc). Resazurin solution was prepared by dissolving powered resazurin (Acros) in phosphate buffered saline (Thermo Fisher) to a final concentration of 100 μg mL^−1^. Solutions of 6a were prepared by adding lyophilized 6a to pure DMSO (EMD Millipore) to a final concentration of 1 mg mL^−1^. Sample fluorescence was measured using a Synergy H4 Hybrid plate reader (BioTek). DCB was obtained from Thermo Fisher. Confocal microscopy was performed with a Leica DMi8 confocal microscope. Image analysis was performed in the LAS X Life Science platform (Leica Microsystems).

### Procedure for the synthesis of ethyl benzotriazolyl acetates 3 and 4

To a suspension of benzotriazole 1 (1 equiv.), ethyl bromoacetate 2 (1.2 equiv.) and K_2_CO_3_ (1.5 equiv.) in THF was sonicated at 50 °C for 5 h. Solvent was then evaporated under reduced pressure and water was added. The mixture was extracted with EtOAc. The combined organic extracts were dried over anhydrous Na_2_SO_4_, filtered and concentrated under reduced pressure. The solid obtained was purified by column chromatography on silica gel using *n*-hexane/EtOAc (3 : 1) as eluent to obtain both isomers 3 and 4.

#### Ethyl 2-(2*H*-benzotriazol-2-yl)acetate 3

(12%) White solid, mp 122–124 °C; ^1^H NMR (500 MHz, CDCl_3_): *δ* 1.27 (t, *J* = 7.1 Hz, 3H), 4.27 (q, *J* = 7.1 Hz, 2H), 5.53 (s, 2H), 7.38–7.42 (m, 2H), 7.86–7.90 (m, 2H); ^13^C NMR (125 MHz, CDCl_3_): *δ* 14.1, 57.2, 62.5, 118.3, 127.0, 145.0, 166.3. HRMS (ESI^+^) *m*/*z* calcd for C_10_H_12_N_3_O_2_ [M + H]^+^ 206.09295, found 206.09214 (literature 48).

#### Ethyl 2-(1*H*-benzotriazol-1-yl)acetate 4

(86%) White solid, mp 83–84 °C; ^1^H NMR (500 MHz, CDCl_3_): *δ* 1.26 (t, *J* = 7.1 Hz, 3H), 4.25 (q, *J* = 7.1 Hz, 2H), 5.42 (s, 2H), 7.38–7.53 (m, 3H), 8.08 (d, *J* = 8.4 Hz, 1H); ^13^C NMR (125 MHz, CDCl_3_): *δ* 14.1, 49.1, 62.3, 109.2, 120.2, 124.1, 127.9, 133.4, 146.0, 166.4. HRMS (ESI^+^) *m*/*z* calcd for C_10_H_12_N_3_O_2_ [M + H]^+^ 206.09295, found 206.09223 (literature 48).

### General procedure for the synthesis of ethyl benzotriazolyl acrylates 6a–d and 7a–d

A solution of the corresponding ethyl benzotriazolyl acetate (3, 4) (1 equiv.), appropriate aromatic aldehydes 5a–d (1 equiv.) and piperidine (2 equiv.) in ethanol (20 mL) was reacted under reflux by conventional heating for 6 h. Solvent was evaporated under reduced pressure. The crude was purified either by column chromatography on silica gel using pure hexane to 70% hexane/EtOAc as eluent or by crystallization under ethanol. The final products were characterized by ^1^H and ^13^C NMR spectroscopy and HRMS. ^1^H and ^13^C-NMR spectra can be found in ESI (Fig. S1–S16†).

### Procedure for the synthesis of ethyl benzotriazolyl acrylates 6c, 7b and 7c using microwave irradiation

In a capped 10 mL MW vessel, the corresponding ethyl benzotriazolyl acetate (3, 4) (1 equiv.), appropriate aromatic aldehydes 5b-c (1 equiv.), piperidine (2 equiv.) and ethanol (4 mL) were mixed. The tube was positioned in the irradiation cavity and the mixture was heated with stirring under microwave irradiation at 100 °C and held for 80 min. The vessel was cooled to room temperature and the residue was dissolved in methanol and then concentrated under reduced pressure. The crude was purified either by column chromatography on silica gel using pure hexane to 70% hexane/EtOAc as eluent or by crystallization under ethanol.

#### Ethyl (*Z*)-2-(2*H*-benzotriazol-2-yl)-3-(4(dimethylamino)phenyl)acrylate 6a

Yellow solid, mp 160–162 °C; ^1^H NMR (300 MHz, CDCl_3_): *δ* 1.24 (t, *J* = 7.1 Hz, 3H), 2.92 (s, 6H), 4.27 (q, *J* = 7.1 Hz, 2H), 6.37 (d, *J* = 9.2 Hz, 2H), 6.44 (d, *J* = 9.2 Hz, 2H), 7.42–7.49 (m, 2H), 7.94–8.00 (m, 2H), 8.01 (s, 1H); ^13^C NMR (75 MHz, CDCl_3_): *δ* 14.2, 39.8, 61.6, 111.5, 118.0, 118.8, 123.9, 126.8, 132.8, 141.1, 145.0, 152.2, 163.9. HRMS (ESI^+^) *m*/*z* calcd for C_19_H_21_N_4_O_2_ [M + H]^+^ 337.16645, found 337.16666.

#### Ethyl (*Z*)-2-(2*H*-benzotriazol-2-yl)-3-(4-(diethylamino)phenyl)acrylate 6b

Yellow solid, mp 142–144 °C; ^1^H NMR (300 MHz, CDCl_3_): *δ* 1.08 (t, *J* = 7.1 Hz, 6H), 1.24 (t, *J* = 7.1 Hz, 3H), 3.28 (q, *J* = 7.1 Hz, 4H), 4.26 (q, *J* = 7.1 Hz, 2H), 6.34 (d, *J* = 9.2 Hz, 2H), 6.41 (d, *J* = 9.2 Hz, 2H), 7.44–7.48 (m, 2H), 7.96–7.98 (m, 1H), 7.99 (s, 1H); ^13^C NMR (125 MHz, CDCl_3_): *δ* 12.6, 14.4, 44.5, 61.7, 111.2, 117.4, 119.0, 123.4, 126.9, 133.3, 141.2, 145.1, 150.2, 164.2. HRMS (ESI^+^) *m*/*z* calcd for C_21_H_25_N_4_O_2_ [M + H]^+^ 365.19775, found 365.19606.

#### Ethyl (*Z*)-2-(2*H*-benzotriazol-2-yl)-3-(4-(diphenylamino)phenyl)acrylate 6c

Yellow oil; ^1^H NMR (300 MHz, CDCl_3_): *δ* 1.24 (t, *J* = 7.1 Hz, 3H), 4.28 (q, *J* = 7.1 Hz, 2H), 6.40 (d, *J* = 8.9 Hz, 2H), 6.68 (d, *J* = 8.9 Hz, 2H), 7.00–7.11 (m, 6H), 7.19–7.29 (m, 4H), 7.36–7.46 (m, 2H), 7.91–7.97 (m, 2H), 8.01 (s, 1H); ^13^C NMR (75 MHz, CDCl_3_): *δ* 14.3, 62.1, 118.3, 118.9, 120.2, 121.6, 122.8, 124.2, 124.8, 125.6, 126.1, 127.3, 129.6, 129.7, 132.3, 140.3, 145.1, 146.3, 151.0, 164.4; HRMS (ESI^+^) *m*/*z* calcd for C_29_H_25_N_4_O_2_ [M + H]^+^ 461.19775, found 461.19840.

#### Ethyl (*Z*)-2-(2*H*-benzotriazol-2-yl)-3-(4-morpholinophenyl)acrylate 6d

Yellow solid, mp 82–84 °C; ^1^H NMR (300 MHz, CDCl_3_): *δ* 1.25 (t, *J* = 7.1 Hz, 3H), 3.16 (t, *J* = 5.1 Hz, 4H), 3.75 (t, *J* = 4.8 Hz, 4H), 4.28 (q, *J* = 7.1 Hz, 2H), 6.49 (d, *J* = 9.1 Hz, 2H), 6.58 (d, *J* = 9.1 Hz, 2H), 7.40–7.52 (m, 2H), 7.91–8.0 (m, 2H), 8.02 (s, 1H). ^13^C NMR (75 MHz, CDCl_3_): *δ* 14.3, 47.4, 62.0, 66.6, 114.1, 118.9, 121.1, 125.7, 127.2, 132.6, 140.6, 145.1, 153.0, 163.7. HRMS (ESI^+^) *m*/*z* calcd for C_21_H_23_N_4_O_3_ [M + H]^+^ 379.17702, found 379.17717.

#### Ethyl (*Z*)-2-(1*H*-benzotriazol-1-yl)-3-(4-(dimethylamino)phenyl)acrylate 7a

Yellow solid, mp 152–154 °C; ^1^H NMR (300 MHz, CDCl_3_): *δ* 1.19 (t, *J* = 7.1 Hz, 3H), 2.91 (s, 6H), 4.23 (q, *J* = 7.1 Hz, 2H), 6.36 (d, *J* = 9.1 Hz, 2H), 6.58 (d, *J* = 9.1 Hz, 2H), 7.29–7.48 (m, 3H), 8.11 (s, 1H), 8.15 (d, *J* = 7.4 Hz, 1H); ^13^C NMR (125 MHz, CDCl_3_): *δ* 14.2, 39.8, 61.5, 110.1, 111.5, 117.7, 118.2, 119.9, 124.1, 128.0, 132.8, 133.6, 142.2, 145.8, 152.1, 164.3. HRMS (ESI^+^) *m*/*z* calcd for C_19_H_21_N_4_O_2_ [M + H]^+^ 337.16645, found 337.16540.

#### Ethyl (*Z*)-2-(1*H*-benzotriazol-1-yl)-3-(4-(diethylamino)phenyl)acrylate 7b

Yellow solid, mp 118–120 °C; ^1^H NMR (300 MHz, CDCl_3_): *δ* 1.07 (t, *J* = 7.1 Hz, 6H), 1.18 (t, *J* = 7.1 Hz, 3H), 3.26 (q, *J* = 7.1 Hz, 4H), 4.22 (q, *J* = 7.1 Hz, 2H), 6.33 (d, *J* = 9.1 Hz, 2H), 6.54 (d, *J* = 9.1 Hz, 2H), 7.31–7.48 (m, 3H), 8.10 (s, 1H), 8.12–8.18 (d, *J* = 8.3 Hz, 1H); ^13^C NMR (75 MHz, CDCl_3_): *δ* 12.5, 14.2, 44.4, 61.5, 110.2, 111.1, 117.0, 117.6, 120.0, 124.1, 128.0, 133.3, 133.7, 142.3, 145.8, 150.1, 164.5. HRMS (ESI^+^) *m*/*z* calcd for C_21_H_25_N_4_O_2_ [M + H]^+^ 365.19775, found 365.19818.

#### Ethyl (*Z*)-2-(1*H*-benzotriazol-1-yl)-3-(4-(diphenylamino)phenyl)acrylate 7c

Yellow oil; ^1^H NMR (300 MHz, CDCl_3_): *δ* 1.19 (t, *J* = 7.1 Hz, 3H), 4.24 (q, *J* = 7.1 Hz, 2H), 6.53 (d, *J* = 8.9 Hz, 2H), 6.68 (d, *J* = 8.9 Hz, 2H), 6.99–7.11 (m, 6H), 7.19–7.28 (m, 4H), 7.31–7.50 (m, 3H), 8.11 (s, 1H), 8.12 (d, *J* = 8.3 Hz, 1H); ^13^C NMR (75 MHz, CDCl_3_): *δ* 14.3, 61.9, 110.1, 120.0, 120.2, 123.0, 124.2, 124.9, 125.7, 126.1, 128.2, 129.7, 132.2, 133.6, 141.4, 145.8, 146.1, 150.8, 164.0. HRMS (ESI^+^) *m*/*z* calcd for C_29_H_25_N_4_O_2_ [M + H]^+^ 461.19775, found 461.19910.

#### Ethyl (*Z*)-2-(1*H*-benzotriazol-1-yl)-3-(4-morpholinophenyl)acrylate 7d

Yellow solid, mp 134–136 °C; ^1^H NMR (500 MHz, CDCl_3_): *δ* 1.20 (t, *J* = 7.1 Hz, 3H), 3.16 (t, *J* = 5.1 Hz, 4H), 3.75 (t, *J* = 5.1 Hz, 4H), 4.24 (q, *J* = 7.1 Hz, 2H), 6.57 (d, *J* = 9.1 Hz, 2H), 6.63 (d, *J* = 9.1 Hz, 2H), 7.31 (d, *J* = 8.1 Hz, 1H), 7.38–7.47 (m, 2H), 8.12 (s, 1H), 8.15 (d, *J* = 8.4 Hz, 1H); ^13^C NMR (125 MHz, CDCl_3_): *δ* 14.3, 47.3, 61.9, 66.5, 110.1, 114.0, 119.8, 120.2, 121.3, 124.2, 128.2, 132.7, 133.6, 141.7, 145.9, 152.9, 164.1. HRMS (ESI^+^) *m*/*z* calcd for C_21_H_23_N_4_O_3_ [M + H]^+^ 379.17702, found 379.17870.

### Single-crystal X-ray diffraction analyses

Compounds 6a, 7a, and 7d were crystallized using slow evaporation in ethyl acetate/hexanes to afford yellow single crystals for X-ray diffraction experiments. High-resolution (0.84 Å) data were collected at 100 K utilizing Cu K_α_ radiation produced by a Bruker-Nonius FR591 rotating anode X-ray source coupled to a MACH3 kappa goniometer and Bruker Apex II CCD detector. The Bruker APEX3 software package was utilized to integrate, scale and correct the obtained data. The structure was solved using dual-space methods in SHELXT^49^ and refined against *F*^2^ on all data by full-matrix least squares with SHELXL-2014 (ref. 50) using established refinement strategies.^51^

### *In vitro* cytotoxicity and cell imaging

For *in vitro* cell toxicity assays, HEK293T cells were co-incubated with 50 μM derivative 6a, and cell viability measured after 24 h by measuring conversion of resazurin to resuforin. For each assay, cells were trypsinized and seeded at 20 000 cells per well in a flat-bottom 96-well optical plate. Cells were seeded and incubated in full media (Dulbecco's Modified Eagle's Medium supplemented with 10% fetal bovine serum) containing 50 μM derivative 6a and 1% DMSO. Vehicle control cells were incubated in full media with 1% DMSO only. After seeding into the optical plate, cells were incubated at 37 °C and 100% humidity with 5% atmospheric CO_2_ for 20 h. Then, a resazurin solution in sterile PBS was added to each well to a final concentration of 25 μg mL^−1^, after which cells were incubated for further 4 h. Well fluorescence was then measured at 590 nm emission and 560 nm excitation using a BioTek Synergy Hybrid plate reader. For the negative control, resazurin solution was added to full media alone and incubated for 4 h before measuring fluorescence. For data analysis, all values were normalized to vehicle control values. All measurements were made in triplicate.

For cell imaging experiments, images were obtained with a Leica DMi8 confocal microscope at 405 nm excitation, with emission filters of 420 ± 30 nm and 545 ± 30 nm for derivative 6a and DCB, respectively. Prior to imaging, HEK293T cells were incubated with 10 μM derivative 6a and 50 μM DCB for 16 h. For modified (pulse-chase) assays, DCB was removed after 12 h by washing cells once with full-serum media (DMEM + FBS), then fresh DMEM + FBS with 10 μM 6a was added, and cells were incubated for further 4 h. Prior to imaging, all samples were washed once with DMEM + FBS, after which cells were incubated in DMEM + FBS only. Cells were imaged live at 37 °C and 100% humidity with 5% atmospheric CO_2_. Image analysis was performed with the LAS X Life Science platform.

## Conflicts of interest

The authors declare that there are no conflicts of interest in this work.

## Supplementary Material

RA-009-C9RA00108E-s001

RA-009-C9RA00108E-s002
