# Supplementary material for: Synthesis and characterization of ethyl benzotriazolyl acrylate-based D–π–A fluorophores for live cell-based imaging applications
Source: RSC Adv. 2019 Mar 18;9(16):8759–67. doi: 10.1039/c9ra00108e (PMC9061700; doi:10.1039/c9ra00108e)
Supplement: RA-009-C9RA00108E-s001 [file RA-009-C9RA00108E-s001.pdf]

Electronic Supplementary Material (ESI) for RSC Advances.
This journal is © The Royal Society of Chemistry 2019

Electronic Supplementary Information (ESI)

Synthesis and characterization of ethyl benzotriazolyl acrylate-based D-π-A fluorophores for live cell-based imaging applications

Ana Sofia Ortega-Villarreal,[a] Eugenio Hernández-Fernández,[a*] Christopher Jensen,[b] Gabriel A. Valdivia-Berroeta,[b] Samuel Garrard,[b] Israel López,[a,c] Stacey J. Smith,[b] Kenneth A. Christensen,[b] Miguel A. Reyes-González,[a] David J. Michaelis[b*]

[a]Universidad Autónoma de Nuevo León, Facultad de Ciencias Químicas, Pedro de Alba s/n, Ciudad Universitaria, 66400, San Nicolás de los Garza, Nuevo León, México

[b]Department of Chemistry and Biochemistry, Brigham Young University, 84602, Provo, Utah, United States

[c]Universidad Autónoma de Nuevo León, UANL, Centro de Investigación en Biotecnología y Nanotecnología (CIBYN), Laboratorio de Nanociencias y Nanotecnología, Autopista al Aeropuerto Internacional Mariano Escobedo Km. 10, Parque de Investigación e Innovación Tecnológica (PIIT) 66629 Apodaca, Nuevo León, México.

Content:

Fig S1. ^{1}H NMR spectrum ($CDCl_3$) of **6a** derivative

Fig S2. ^{13}C NMR spectrum ($CDCl_3$) of **6a** derivative

Fig S3. ^{1}H NMR spectrum ($CDCl_3$) of **6b** derivative

Fig S4. ^{13}C NMR spectrum ($CDCl_3$) of **6b** derivative

Fig S5. ^{1}H NMR spectrum ($CDCl_3$) of **6c** derivative

Fig S6. ^{13}C NMR spectrum ($CDCl_3$) of **6c** derivative

Fig S7. ^{1}H NMR spectrum ($CDCl_3$) of **6d** derivative

Fig S8. ^{13}C NMR spectrum ($CDCl_3$) of **6d** derivative

Fig S9. ^{1}H NMR spectrum ($CDCl_3$) of **7a** derivative

Fig S10. ^{13}C NMR spectrum ($CDCl_3$) of **7a** derivative

Fig S11. ^{1}H NMR spectrum ($CDCl_3$) of **7b** derivative

Fig S12. ^{13}C NMR spectrum ($CDCl_3$) of **7b** derivative

Fig S13. ^{1}H NMR spectrum ($CDCl_3$) of **7c** derivative

Fig S14. ^{13}C NMR spectrum ($CDCl_3$) of **7c** derivative

Fig S15. ^{1}H NMR spectrum ($CDCl_3$) of **7d** derivative

Fig S16. ^{13}C NMR spectrum ($CDCl_3$) of **7d** derivative

Fig S17. Single-crystal X-ray analysis of **6a** derivative (CCDC 1882468)

Table S1. Atomic coordinates (x 10^4) and equivalent isotropic displacement parameters (Å^{2}x 10^3) for **6a** derivative. U(eq) is defined as one third of the trace of the orthogonalized U^{ij} tensor.

Table S2. Bond lengths [Å] and angles [°] of **6a** derivative.

Table S3. Anisotropic displacement parameters (Å^{2}x 10^3) for **6a** derivative. The anisotropic displacement factor exponent takes the form: $-2\pi^2$[h^2 a^{*2} U^{11} + ... + 2 h k a* b* U^{12}].

Table S4. Hydrogen coordinates (x 10^4) and isotropic displacement parameters (Å^{2}x 10^3) for **6a** derivative.

Fig S18. Single-crystal X-ray analysis of **7a** derivative (CCDC 1867840)

Table S5. Atomic coordinates (x 10^4) and equivalent isotropic displacement parameters ($Å^2$x 10^3) for **7a** derivative. U(eq) is defined as one third of the trace of the orthogonalized U^{ij} tensor.

Table S6. Bond lengths [Å] and angles [°] for **7a** derivative.

Table S7. Anisotropic displacement parameters ($Å^2$x 10^3) for **7a** derivative. The anisotropic displacement factor exponent takes the form: $-2\pi^2[h^2 a^{*2}U^{11} + ... + 2 h k a^* b^* U^{12}]$.

Table S8. Hydrogen coordinates (x 10^4) and isotropic displacement parameters ($Å^2$x 10^3) for **7a** derivative.

Fig S19. Single-crystal X-ray analysis of **7d** derivative (CCDC 1882467)

Table S9. Atomic coordinates (x 10^4) and equivalent isotropic displacement parameters ($Å^2$x 10^3) for **7d** derivative. U(eq) is defined as one third of the trace of the orthogonalized U^{ij} tensor.

Table S10. Bond lengths [Å] and angles [°] for **7d** derivative.

Table S11. Anisotropic displacement parameters ($Å^2$x 10^3) for **7d** derivative. The anisotropic displacement factor exponent takes the form: $-2\pi^2[h^2 a^{*2}U^{11} + ... + 2 h k a^* b^* U^{12}]$.

Table S12. Hydrogen coordinates (x10^4) and isotropic displacement parameters ($Å^2$x 10^3) for **7d** derivatives.

Fig S20. Absorption spectra of **6b** derivative

Fig S21. Absorption and emission spectra of **6a** derivative in (solid line) methanol and (dot line) DMSO solution

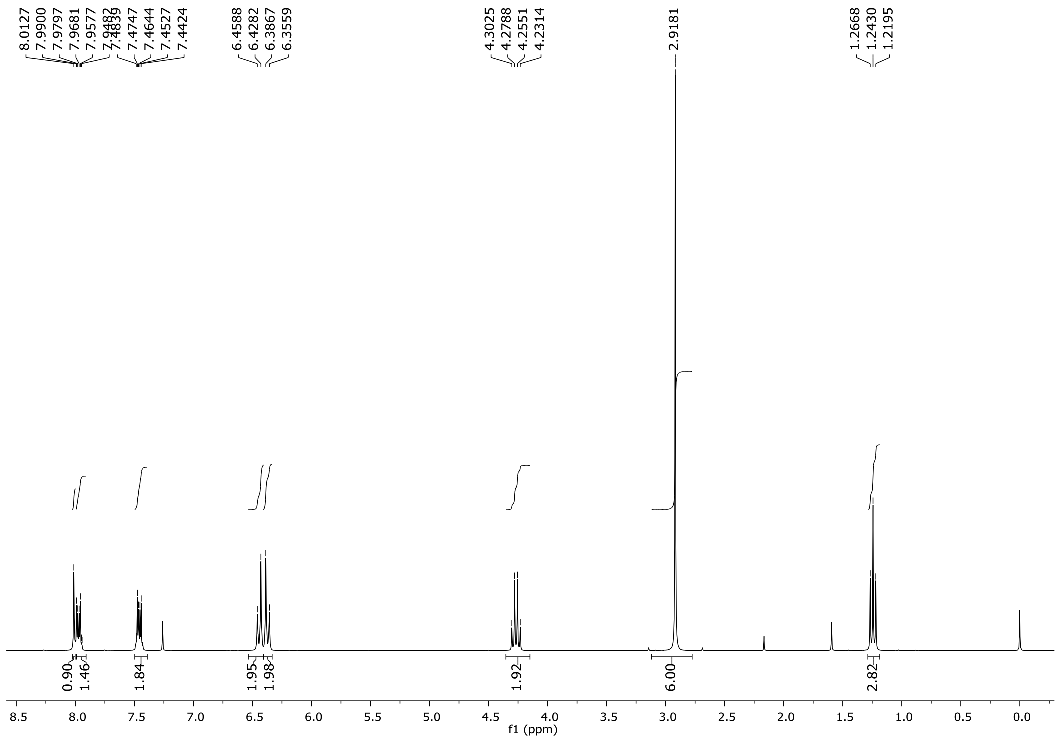

Fig S3. ^{1}H NMR spectrum ($CDCl_3$) of **6a** derivative

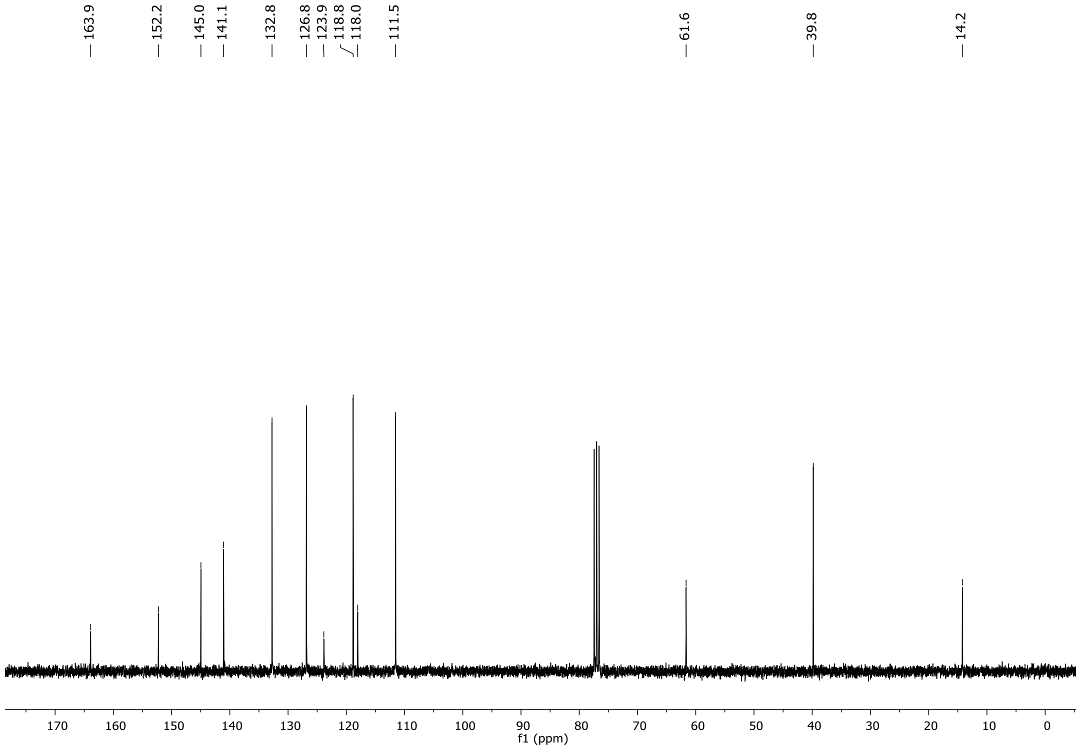

Fig S4. ^{13}C NMR spectrum ($CDCl_3$) of **6a** derivative

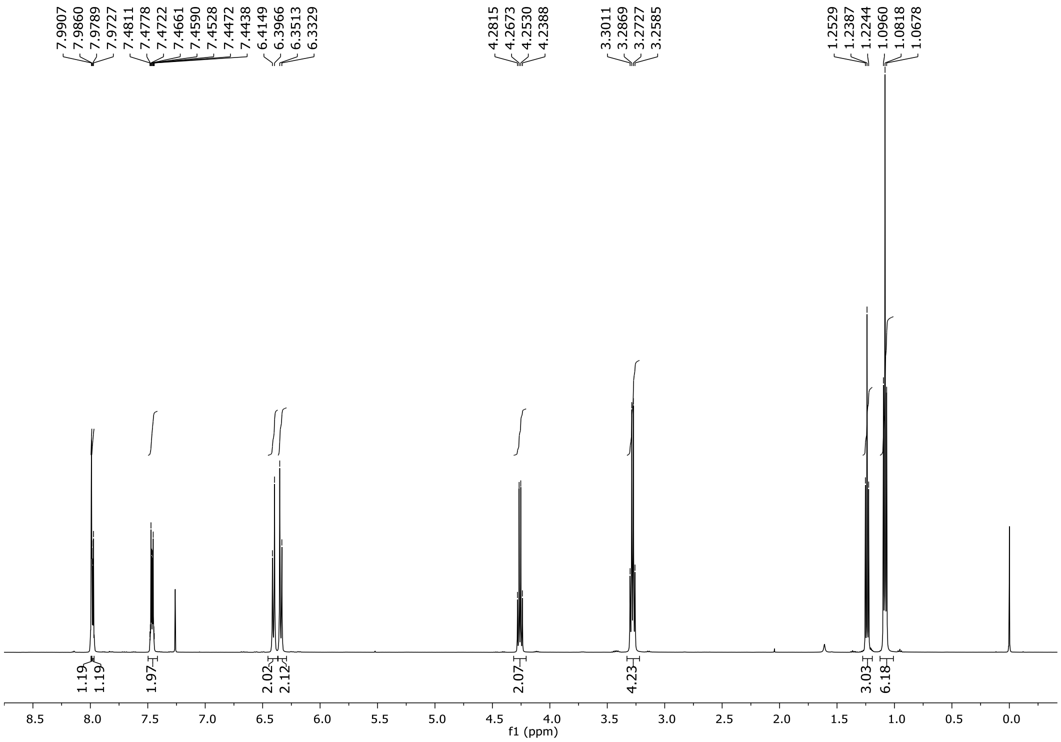

Fig S5. ^{1}H NMR spectrum ($CDCl_3$) of **6b** derivative

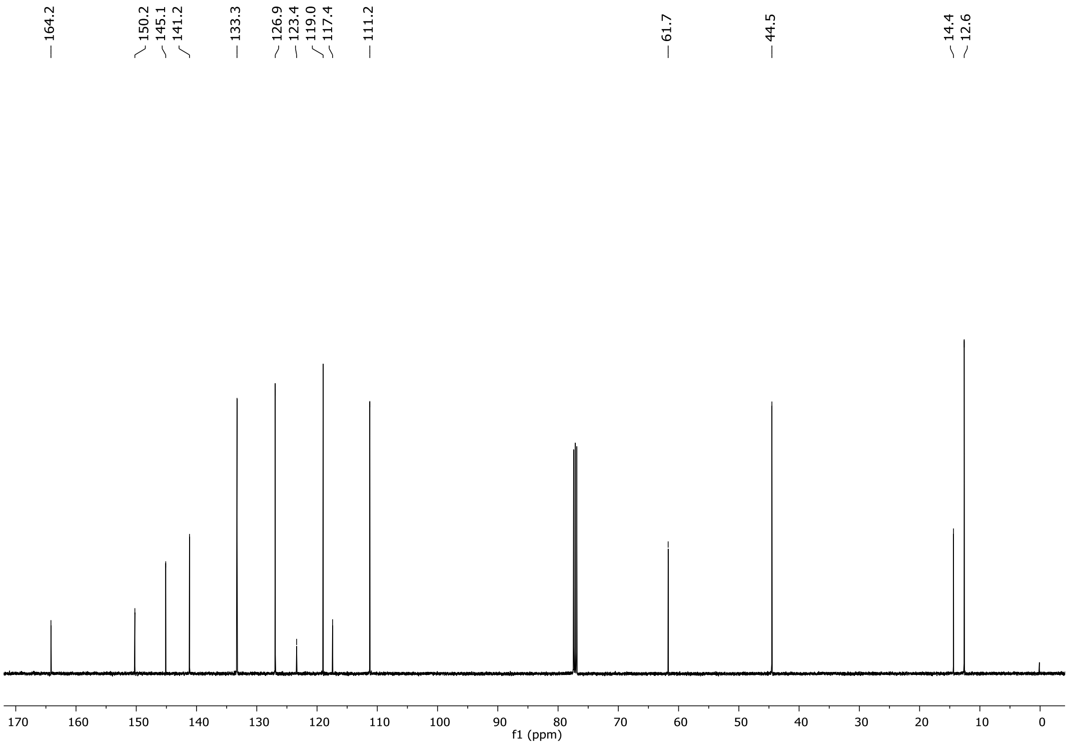

Fig S6. ^{13}C NMR spectrum ($CDCl_3$) of **6b** derivative

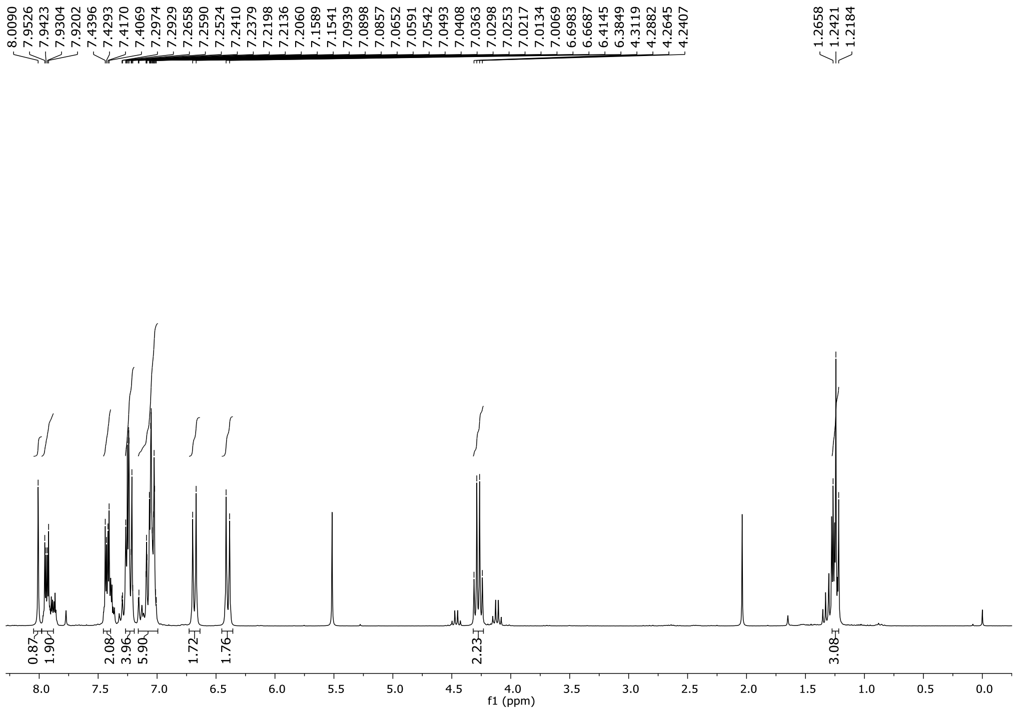

Fig S7. ^{1}H NMR spectrum ($CDCl_3$) of **6c** derivative

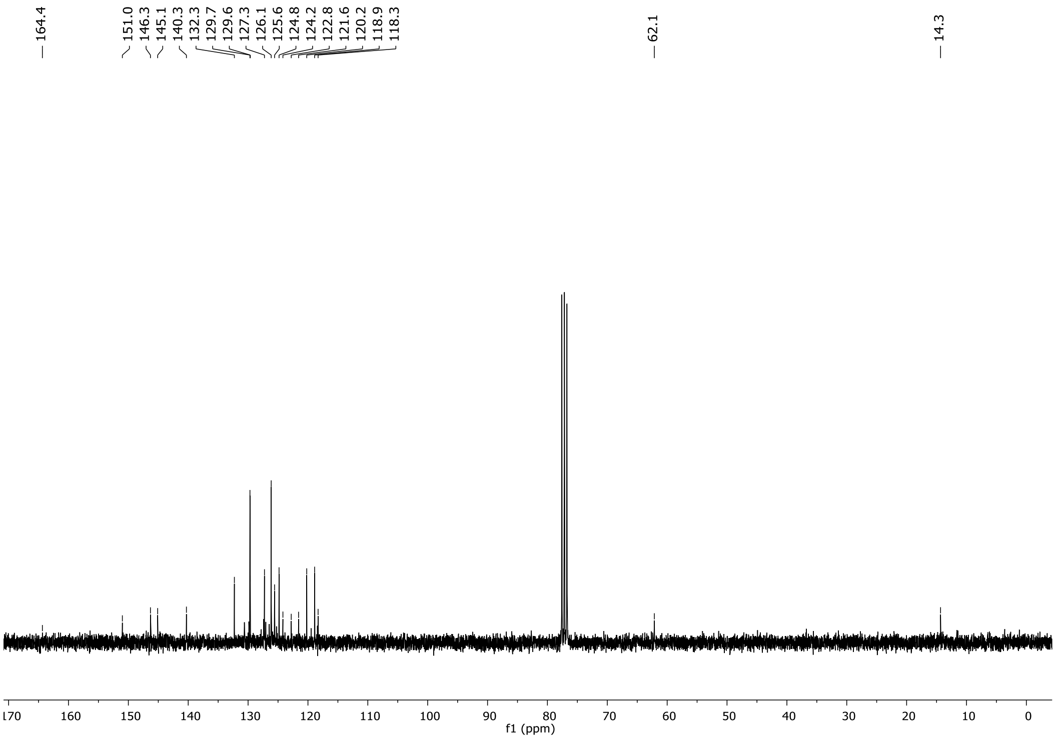

Fig S8. ^{13}C NMR spectrum ($CDCl_3$) of **6c** derivative

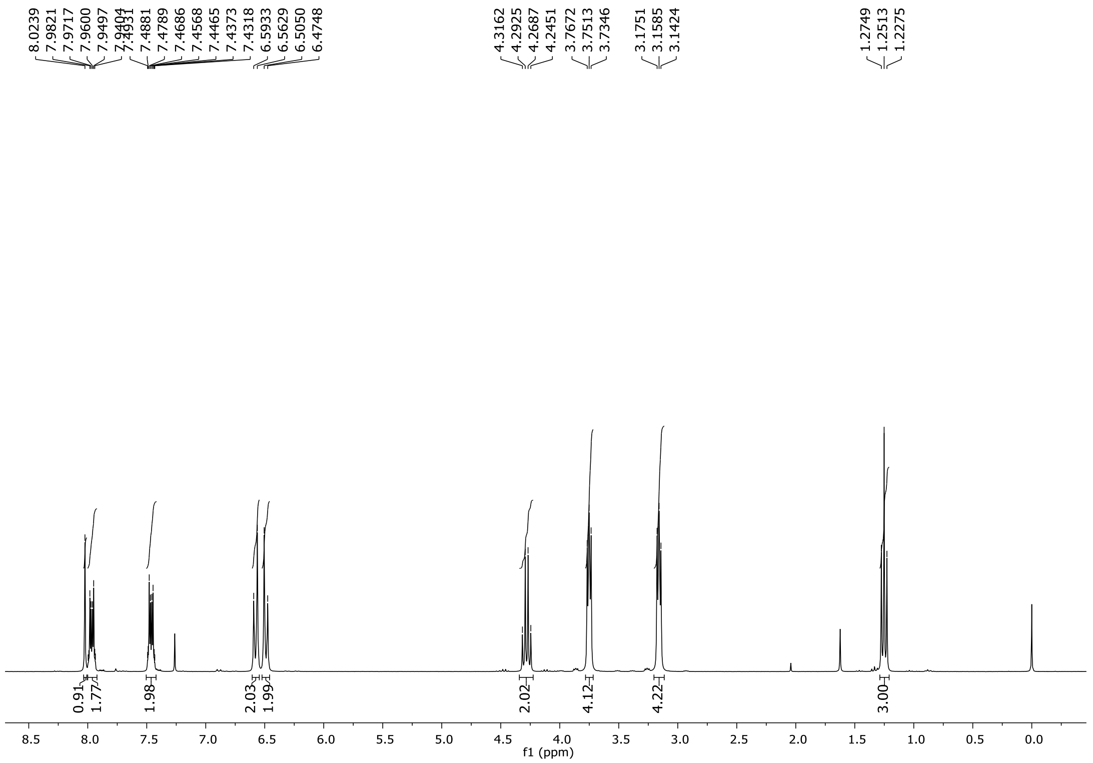

Fig S9. ^{1}H NMR spectrum ($CDCl_3$) of **6d** derivative

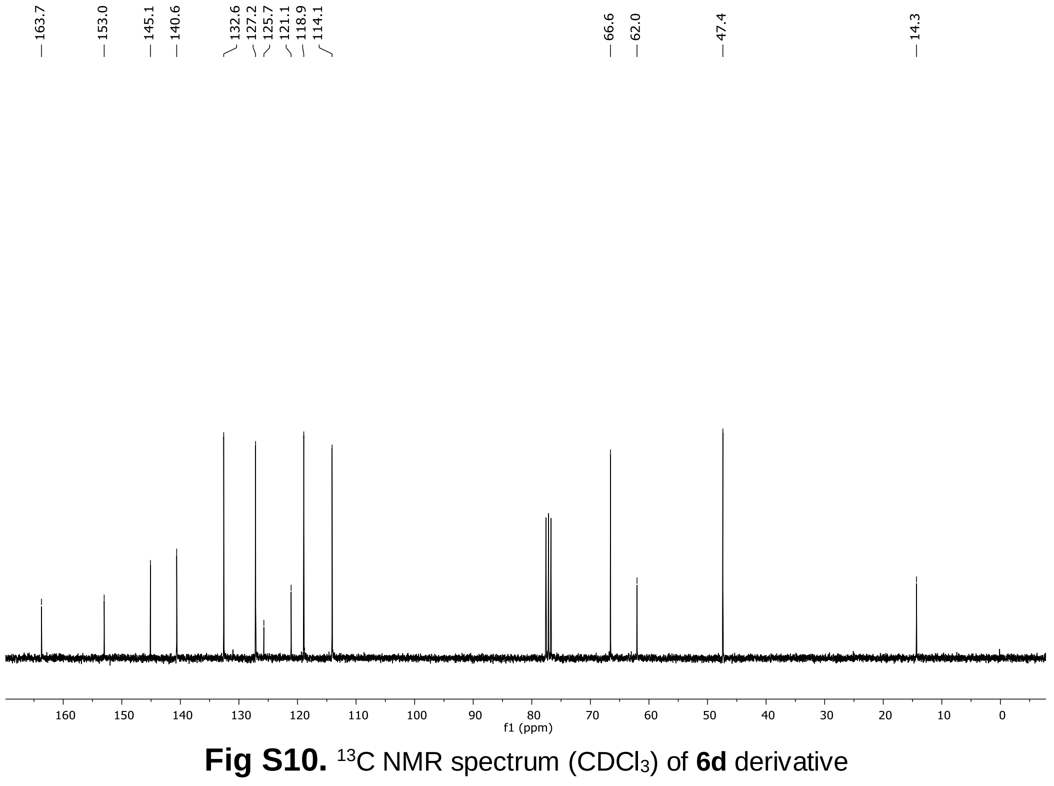

Fig S10. ^{13}C NMR spectrum ($CDCl_3$) of **6d** derivative

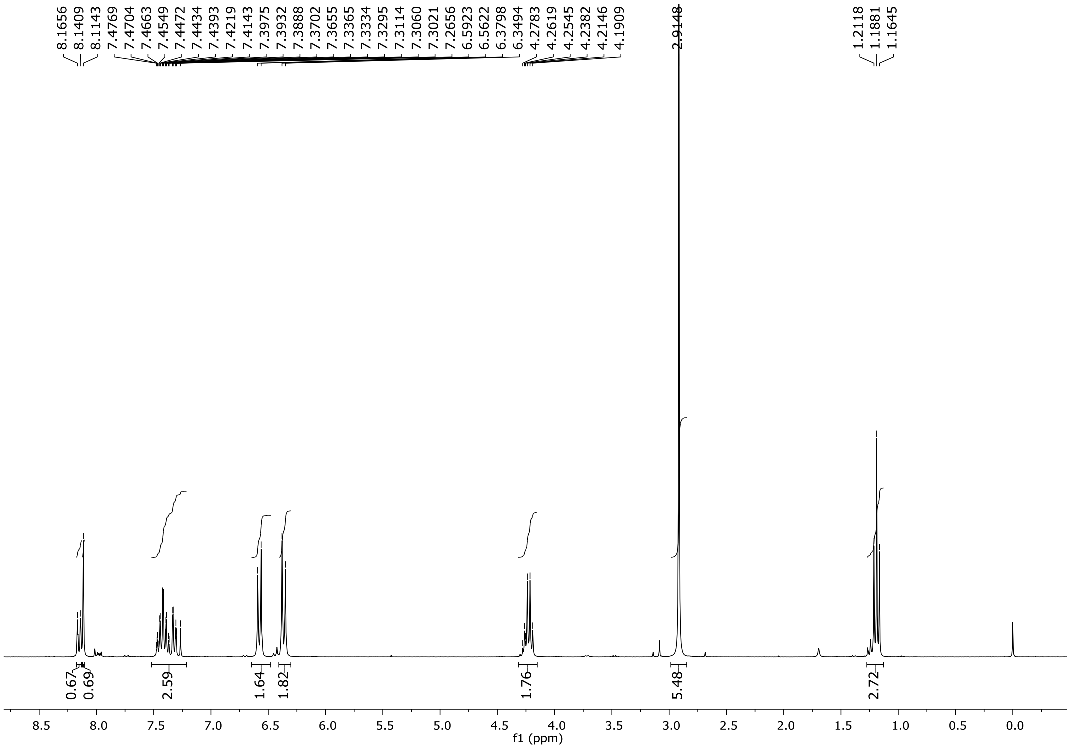

Fig S11. ^{1}H NMR spectrum ($CDCl_3$) of **7a** derivative

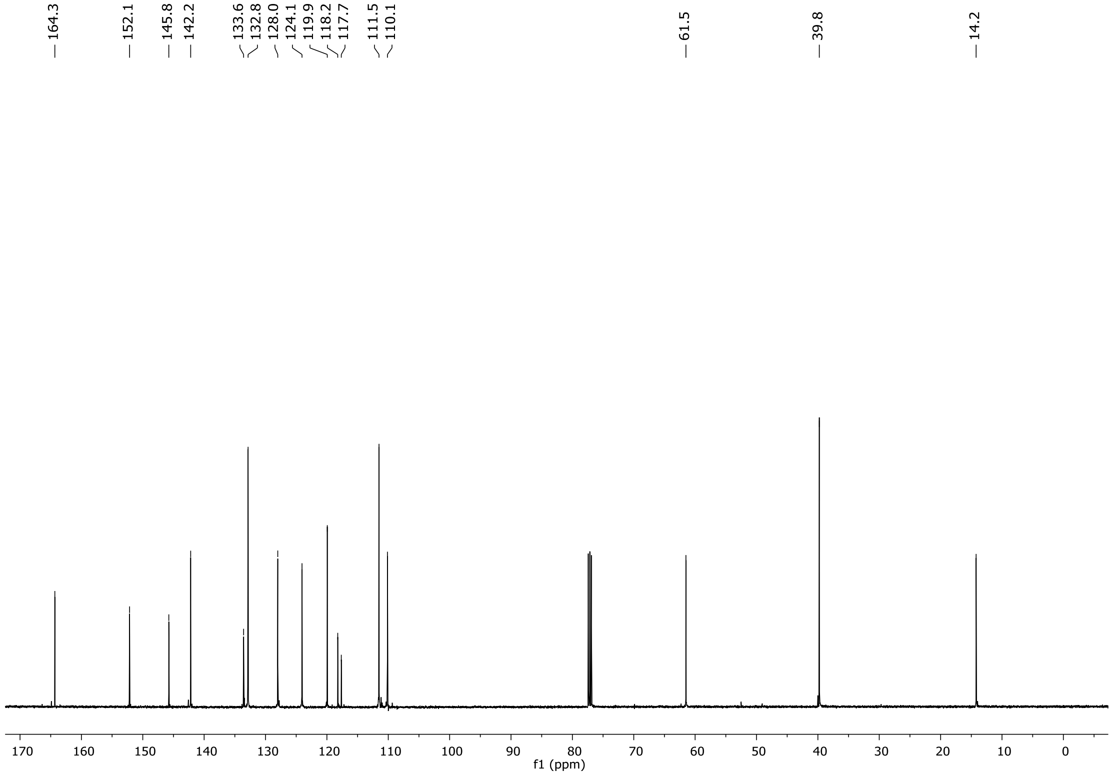

Fig S12. ^{13}C NMR spectrum ($CDCl_3$) of **7a** derivative

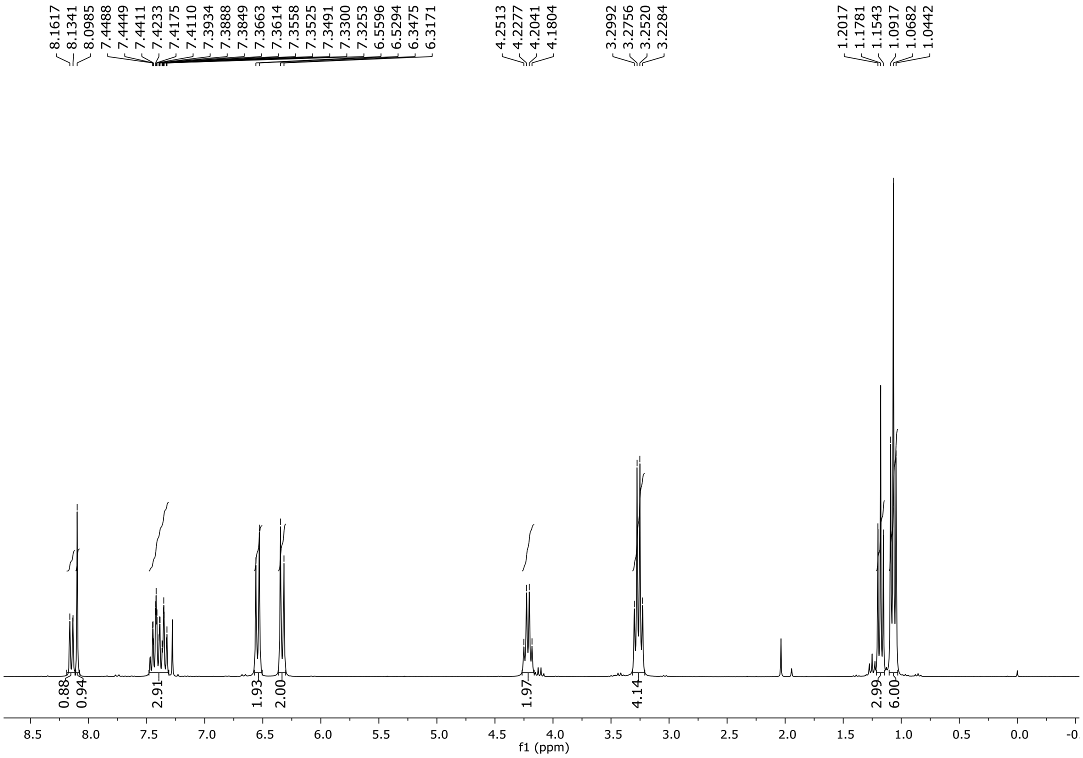

Fig S13. ^{1}H NMR spectrum ($CDCl_3$) of **7b** derivative

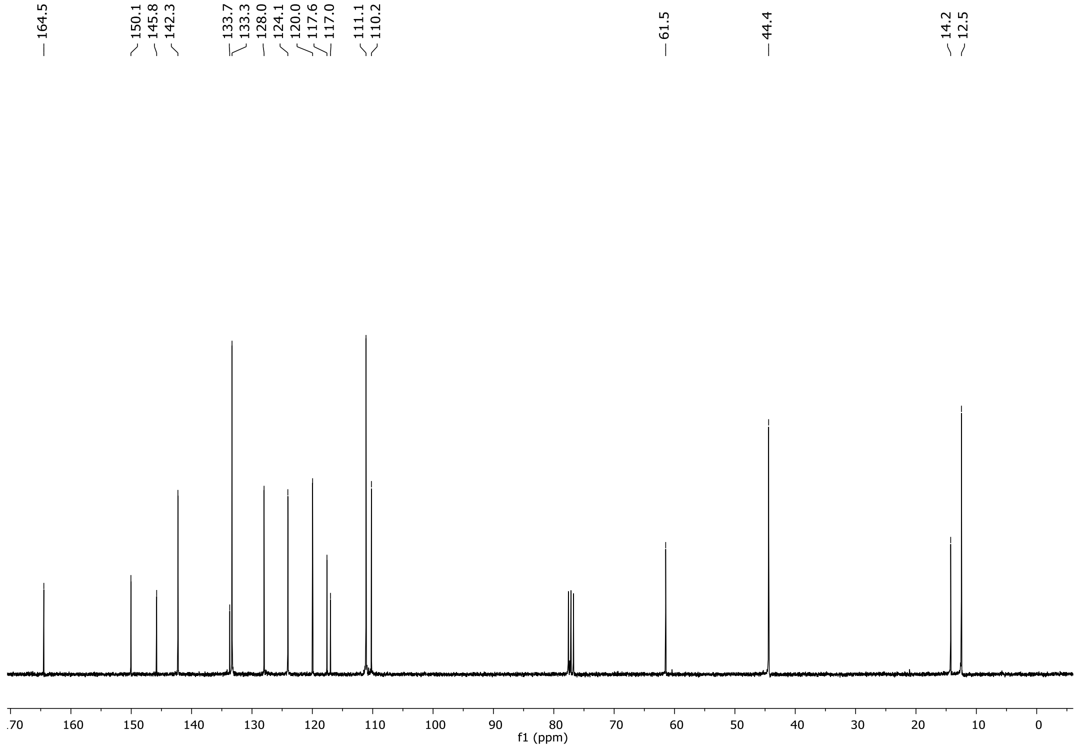

Fig S14. ^{13}C NMR spectrum ($CDCl_3$) of **7b** derivative

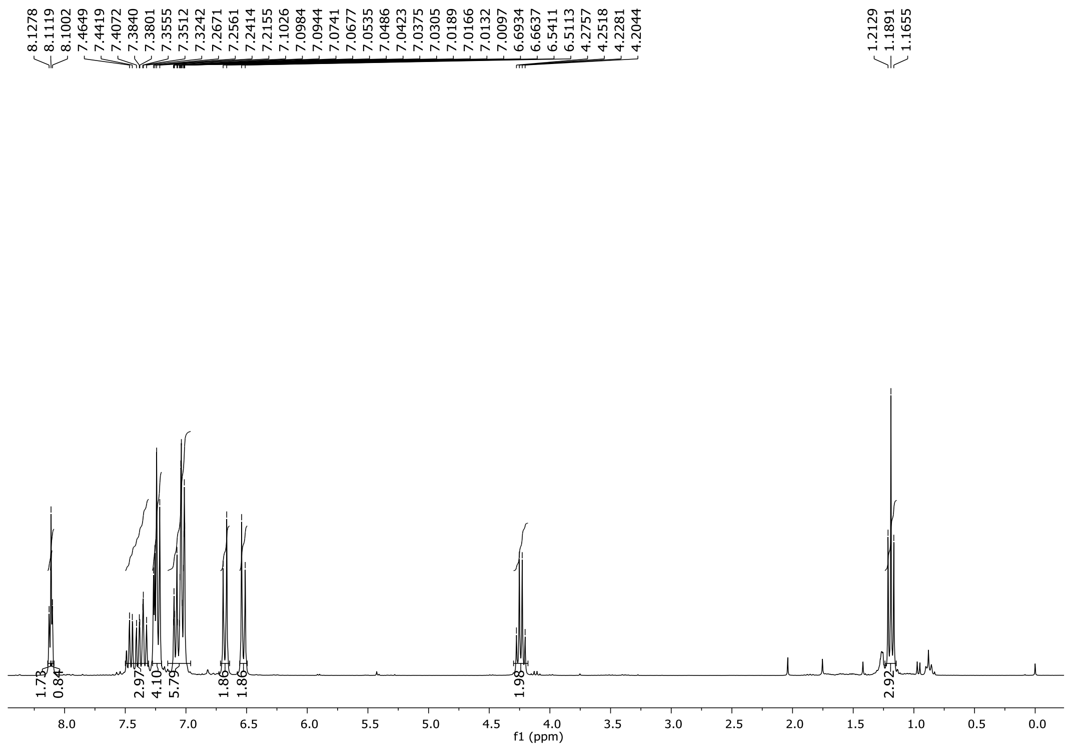

Fig S15. ^{1}H NMR spectrum ($CDCl_3$) of **7c** derivative

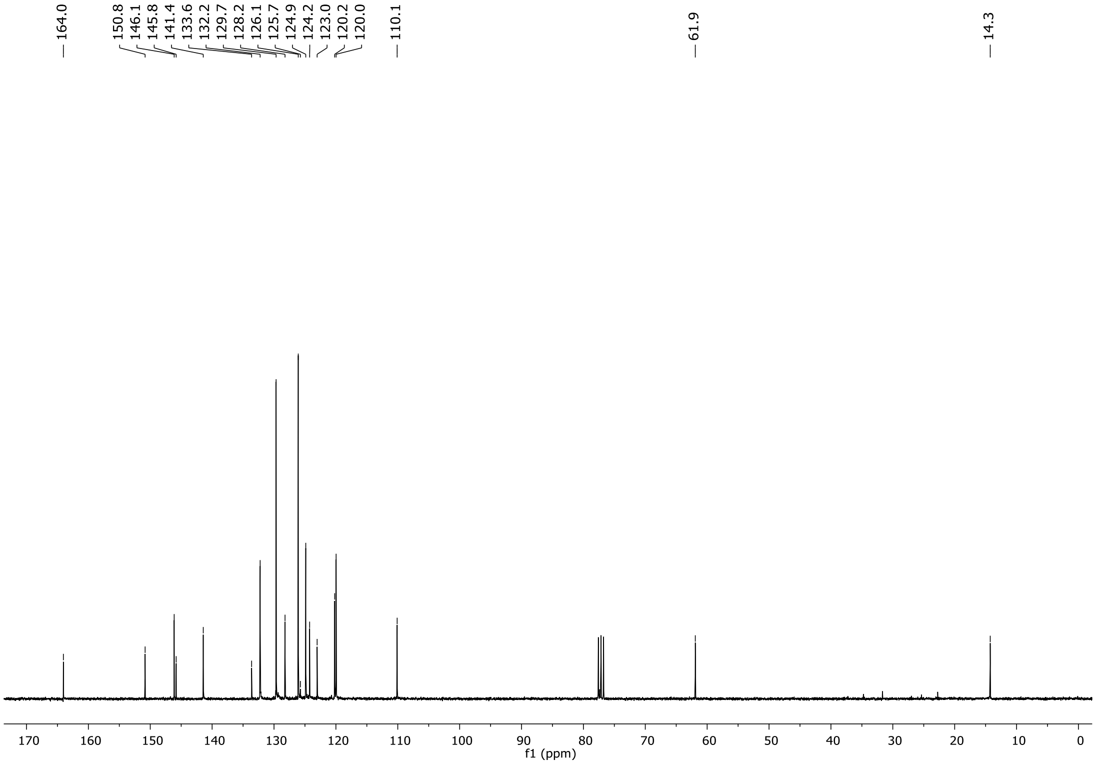

Fig S16. ^{13}C NMR spectrum ($CDCl_3$) of **7c** derivative

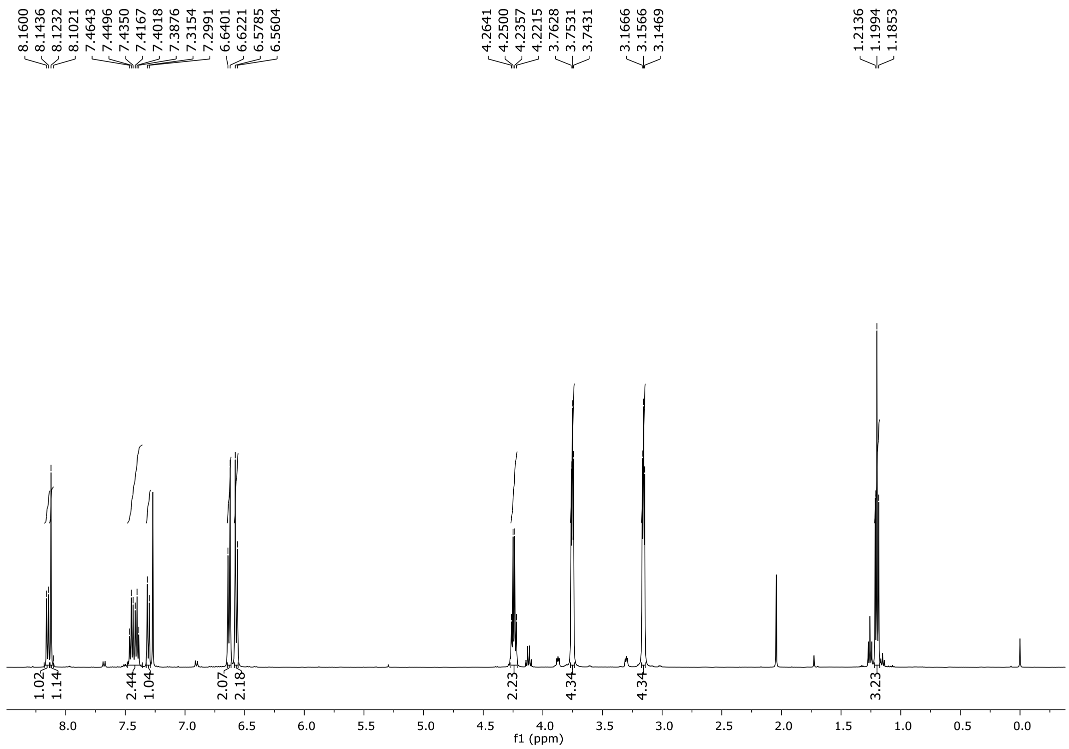

Fig S17. ^{1}H NMR spectrum ($CDCl_3$) of **7d** derivative

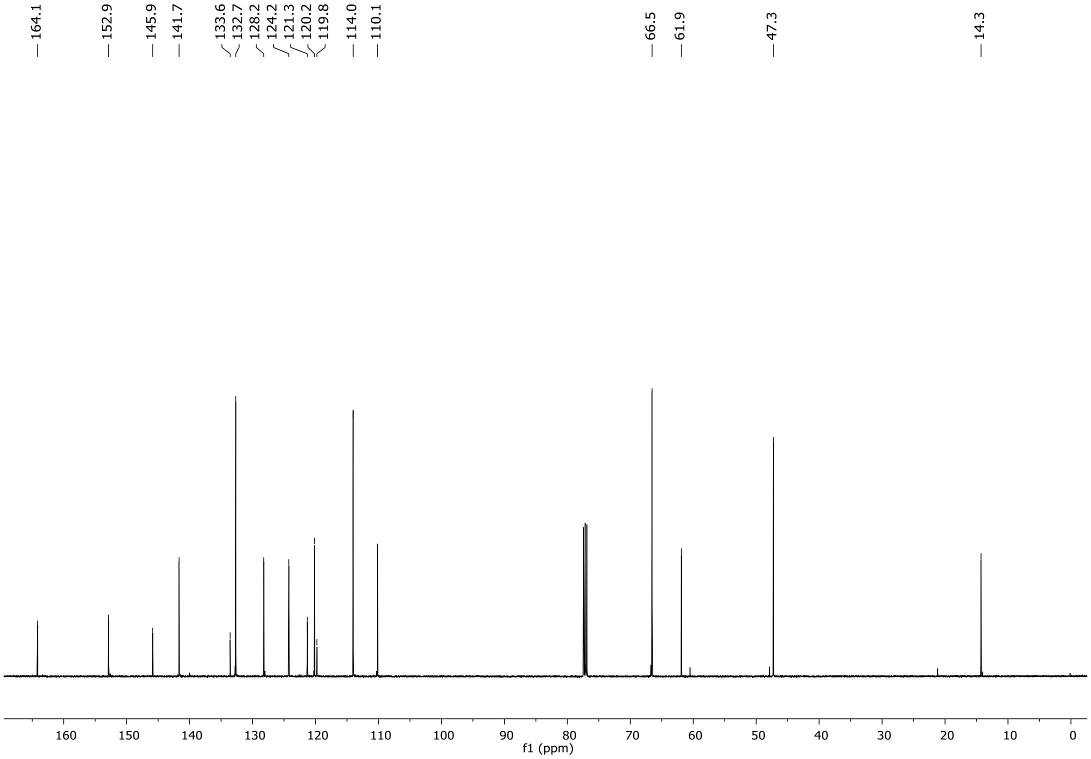

Fig S18. ^{13}C NMR spectrum ($CDCl_3$) of **7d** derivative

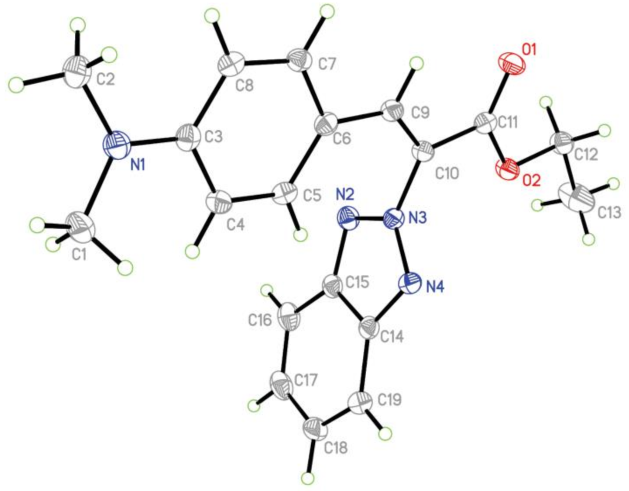

Fig S19. Single-crystal X-ray analysis of **6a** derivative (CCDC 1882468)

Table S1. Atomic coordinates (x 10^4) and equivalent isotropic displacement parameters (Å^{2}x 10^3) for **6a** derivative. U(eq) is defined as one third of the trace of the orthogonalized U^{ij} tensor.

	x	y	z	U(eq)
O(1)	3490(1)	-1103(1)	5189(1)	24(1)
C(1)	5596(2)	8781(2)	7024(1)	27(1)
N(1)	5895(1)	8112(1)	6469(1)	23(1)
C(2)	6864(2)	9014(2)	6132(1)	26(1)
N(2)	576(1)	1913(1)	6052(1)	20(1)
O(2)	1700(1)	-1578(1)	5761(1)	21(1)
N(3)	1886(1)	1272(1)	6260(1)	16(1)
C(3)	5417(1)	6624(2)	6301(1)	18(1)
N(4)	2179(1)	1073(1)	6839(1)	18(1)
C(4)	4364(1)	5792(2)	6595(1)	20(1)
C(7)	5456(1)	4316(2)	5667(1)	17(1)
C(6)	4408(1)	3493(2)	5954(1)	17(1)
C(5)	3872(1)	4291(2)	6423(1)	19(1)
C(9)	4001(1)	1905(2)	5742(1)	18(1)
C(8)	5933(1)	5836(2)	5824(1)	18(1)
C(10)	2941(1)	881(2)	5868(1)	17(1)
C(11)	2762(1)	-685(2)	5568(1)	18(1)
C(12)	1418(2)	-3093(2)	5456(1)	22(1)
C(13)	210(2)	-3917(2)	5727(1)	45(1)
C(14)	930(1)	1655(1)	7032(1)	18(1)
C(15)	-62(1)	2166(1)	6544(1)	18(1)
C(16)	-1460(2)	2844(2)	6612(1)	25(1)
C(17)	-1798(2)	2988(2)	7169(1)	27(1)
C(18)	-791(2)	2494(2)	7663(1)	27(1)
C(19)	563(2)	1825(2)	7607(1)	24(1)

Table S2. Bond lengths [Å] and angles [°] of **6a** derivative.

O(1)-C(11)	1.2074(15)
C(1)-N(1)	1.4528(16)
C(1)-H(1A)	0.9800
C(1)-H(1B)	0.9800
C(1)-H(1C)	0.9800
N(1)-C(3)	1.3599(17)
N(1)-C(2)	1.4513(17)
C(2)-H(2A)	0.9800
C(2)-H(2B)	0.9800
C(2)-H(2C)	0.9800
N(2)-N(3)	1.3309(14)
N(2)-C(15)	1.3490(16)
O(2)-C(11)	1.3358(15)
O(2)-C(12)	1.4575(15)
N(3)-N(4)	1.3371(14)
N(3)-C(10)	1.4304(15)
C(3)-C(8)	1.4094(17)
C(3)-C(4)	1.4176(17)
N(4)-C(14)	1.3535(16)
C(4)-C(5)	1.3756(18)
C(4)-H(4)	0.9500
C(7)-C(8)	1.3776(18)
C(7)-C(6)	1.4008(17)
C(7)-H(7)	0.9500
C(6)-C(5)	1.4076(17)
C(6)-C(9)	1.4485(17)
C(5)-H(5)	0.9500
C(9)-C(10)	1.3426(17)
C(9)-H(9)	0.9500
C(8)-H(8)	0.9500
C(10)-C(11)	1.4830(17)
C(12)-C(13)	1.4909(19)
C(12)-H(12A)	0.9900
C(12)-H(12B)	0.9900

C(13)-H(13A)	0.9800
C(13)-H(13B)	0.9800
C(13)-H(13C)	0.9800
C(14)-C(19)	1.4118(18)
C(14)-C(15)	1.4131(17)
C(15)-C(16)	1.4094(18)
C(16)-C(17)	1.362(2)
C(16)-H(16)	0.9500
C(17)-C(18)	1.425(2)
C(17)-H(17)	0.9500
C(18)-C(19)	1.3638(19)
C(18)-H(18)	0.9500
C(19)-H(19)	0.9500

N(1)-C(1)-H(1A)	109.5
N(1)-C(1)-H(1B)	109.5
H(1A)-C(1)-H(1B)	109.5
N(1)-C(1)-H(1C)	109.5
H(1A)-C(1)-H(1C)	109.5
H(1B)-C(1)-H(1C)	109.5
C(3)-N(1)-C(2)	121.03(11)
C(3)-N(1)-C(1)	121.19(11)
C(2)-N(1)-C(1)	117.43(11)
N(1)-C(2)-H(2A)	109.5
N(1)-C(2)-H(2B)	109.5
H(2A)-C(2)-H(2B)	109.5
N(1)-C(2)-H(2C)	109.5
H(2A)-C(2)-H(2C)	109.5
H(2B)-C(2)-H(2C)	109.5
N(3)-N(2)-C(15)	102.40(10)
C(11)-O(2)-C(12)	114.53(9)
N(2)-N(3)-N(4)	117.63(10)
N(2)-N(3)-C(10)	119.78(10)
N(4)-N(3)-C(10)	122.54(10)
N(1)-C(3)-C(8)	121.87(11)
N(1)-C(3)-C(4)	121.44(11)

C(8)-C(3)-C(4)	116.69(11)
N(3)-N(4)-C(14)	102.44(10)
C(5)-C(4)-C(3)	121.52(11)
C(5)-C(4)-H(4)	119.2
C(3)-C(4)-H(4)	119.2
C(8)-C(7)-C(6)	122.55(11)
C(8)-C(7)-H(7)	118.7
C(6)-C(7)-H(7)	118.7
C(7)-C(6)-C(5)	116.46(11)
C(7)-C(6)-C(9)	116.82(11)
C(5)-C(6)-C(9)	126.71(11)
C(4)-C(5)-C(6)	121.75(11)
C(4)-C(5)-H(5)	119.1
C(6)-C(5)-H(5)	119.1
C(10)-C(9)-C(6)	132.21(11)
C(10)-C(9)-H(9)	113.9
C(6)-C(9)-H(9)	113.9
C(7)-C(8)-C(3)	120.99(11)
C(7)-C(8)-H(8)	119.5
C(3)-C(8)-H(8)	119.5
C(9)-C(10)-N(3)	122.53(11)
C(9)-C(10)-C(11)	120.25(11)
N(3)-C(10)-C(11)	117.10(10)
O(1)-C(11)-O(2)	123.86(11)
O(1)-C(11)-C(10)	123.79(11)
O(2)-C(11)-C(10)	112.34(10)
O(2)-C(12)-C(13)	107.07(10)
O(2)-C(12)-H(12A)	110.3
C(13)-C(12)-H(12A)	110.3
O(2)-C(12)-H(12B)	110.3
C(13)-C(12)-H(12B)	110.3
H(12A)-C(12)-H(12B)	108.6
C(12)-C(13)-H(13A)	109.5
C(12)-C(13)-H(13B)	109.5
H(13A)-C(13)-H(13B)	109.5
C(12)-C(13)-H(13C)	109.5

H(13A)-C(13)-H(13C)	109.5
H(13B)-C(13)-H(13C)	109.5
N(4)-C(14)-C(19)	130.40(12)
N(4)-C(14)-C(15)	108.43(11)
C(19)-C(14)-C(15)	121.15(11)
N(2)-C(15)-C(16)	129.68(12)
N(2)-C(15)-C(14)	109.10(11)
C(16)-C(15)-C(14)	121.21(12)
C(17)-C(16)-C(15)	116.77(13)
C(17)-C(16)-H(16)	121.6
C(15)-C(16)-H(16)	121.6
C(16)-C(17)-C(18)	122.08(12)
C(16)-C(17)-H(17)	119.0
C(18)-C(17)-H(17)	119.0
C(19)-C(18)-C(17)	122.14(13)
C(19)-C(18)-H(18)	118.9
C(17)-C(18)-H(18)	118.9
C(18)-C(19)-C(14)	116.64(12)
C(18)-C(19)-H(19)	121.7
C(14)-C(19)-H(19)	121.7

Symmetry transformations used to generate equivalent atoms:

Table S3. Anisotropic displacement parameters ($Å^2$x 10^3) for **6a** derivative. The anisotropic displacement factor exponent takes the form: $-2\pi^2[h^2 a^{*2} U^{11} + ... + 2 h k a^* b^* U^{12}]$

	U^{11}	U^{22}	U^{33}	U^{23}	U^{13}	U^{12}
O(1)	28(1)	22(1)	24(1)	-5(1)	12(1)	-4(1)
C(1)	32(1)	22(1)	26(1)	-6(1)	5(1)	-2(1)
N(1)	27(1)	20(1)	22(1)	-3(1)	4(1)	-4(1)
C(2)	28(1)	20(1)	29(1)	-2(1)	5(1)	-5(1)
N(2)	19(1)	19(1)	20(1)	-1(1)	1(1)	1(1)
O(2)	23(1)	17(1)	23(1)	-4(1)	8(1)	-4(1)
N(3)	17(1)	15(1)	17(1)	1(1)	3(1)	-1(1)
C(3)	18(1)	18(1)	18(1)	1(1)	-2(1)	1(1)
N(4)	20(1)	19(1)	17(1)	2(1)	4(1)	1(1)
C(4)	24(1)	21(1)	16(1)	-1(1)	3(1)	1(1)
C(7)	17(1)	20(1)	15(1)	0(1)	1(1)	2(1)
C(6)	18(1)	18(1)	16(1)	2(1)	1(1)	1(1)
C(5)	20(1)	20(1)	17(1)	2(1)	4(1)	-1(1)
C(9)	19(1)	19(1)	15(1)	1(1)	2(1)	2(1)
C(8)	17(1)	21(1)	17(1)	3(1)	2(1)	-1(1)
C(10)	18(1)	18(1)	16(1)	0(1)	4(1)	1(1)
C(11)	18(1)	19(1)	17(1)	2(1)	2(1)	0(1)
C(12)	29(1)	18(1)	21(1)	-5(1)	4(1)	-5(1)
C(13)	50(1)	35(1)	54(1)	-19(1)	28(1)	-23(1)
C(14)	19(1)	14(1)	21(1)	1(1)	5(1)	-1(1)
C(15)	18(1)	14(1)	22(1)	-1(1)	3(1)	-2(1)
C(16)	20(1)	21(1)	33(1)	-2(1)	2(1)	2(1)
C(17)	22(1)	22(1)	40(1)	-4(1)	12(1)	2(1)
C(18)	34(1)	23(1)	28(1)	-1(1)	16(1)	1(1)
C(19)	30(1)	22(1)	21(1)	2(1)	8(1)	2(1)

Table S4. Hydrogen coordinates (x 10^4) and isotropic displacement parameters ($Å^2$x 10^3) for **6a** derivative.

	x	y	z	U(eq)
H(1A)	6277	9676	7130	40
H(1B)	5749	7959	7328	40
H(1C)	4561	9160	6989	40
H(2A)	6487	8939	5715	38
H(2B)	7879	8577	6200	38
H(2C)	6879	10135	6254	38
H(4)	3990	6283	6919	24
H(7)	5852	3807	5352	21
H(5)	3152	3779	6627	23
H(9)	4596	1514	5461	21
H(8)	6620	6360	5608	22
H(12A)	2336	-3753	5498	27
H(12B)	1101	-2907	5035	27
H(13A)	-716	-3297	5653	67
H(13B)	503	-4011	6150	67
H(13C)	49	-4983	5556	67
H(16)	-2134	3183	6285	30
H(17)	-2735	3432	7231	33
H(18)	-1071	2635	8043	32
H(19)	1225	1490	7938	29

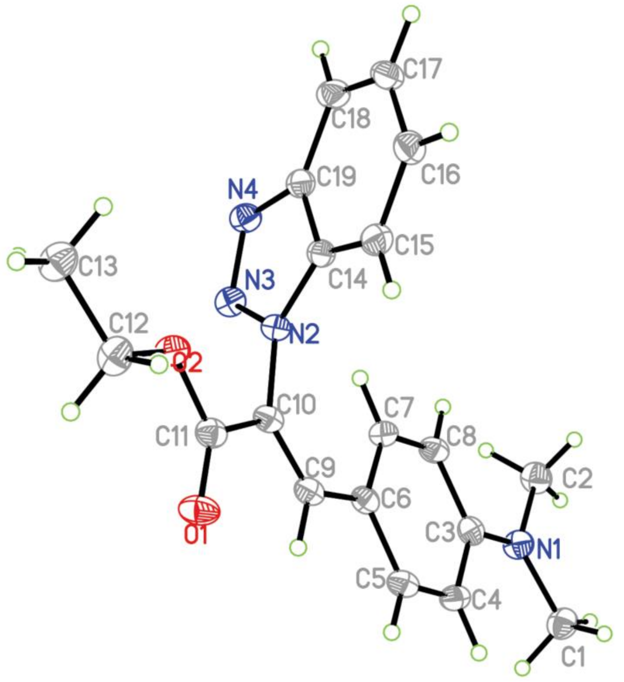

Fig S20. Single-crystal X-ray analysis of **7a** derivative (CCDC 1867840)

Table S5. Atomic coordinates (x 10^4) and equivalent isotropic displacement parameters (Å^{2}x 10^3) for **7a** derivative. U(eq) is defined as one third of the trace of the orthogonalized U^{ij} tensor.

	x	y	z	U(eq)
O(1)	7352(1)	847(1)	2892(1)	25(1)
N(1)	4962(1)	9049(1)	1703(1)	22(1)
C(1)	4209(1)	8836(2)	1310(1)	25(1)
N(2)	8301(1)	4700(1)	3093(1)	17(1)
O(2)	8684(1)	1841(1)	3338(1)	22(1)
C(2)	5328(1)	10595(2)	1804(1)	26(1)
N(3)	8053(1)	5671(1)	3429(1)	20(1)
C(3)	5419(1)	7813(2)	1924(1)	19(1)
N(4)	8833(1)	6537(1)	3558(1)	21(1)
C(4)	5103(1)	6301(2)	1814(1)	20(1)
C(5)	5562(1)	5070(2)	2042(1)	20(1)
C(6)	6372(1)	5236(2)	2389(1)	18(1)
C(7)	6685(1)	6745(2)	2499(1)	20(1)
C(8)	6224(1)	7994(2)	2278(1)	20(1)
C(9)	6799(1)	3847(2)	2597(1)	19(1)
C(10)	7616(1)	3552(1)	2898(1)	18(1)
C(11)	7850(1)	1945(2)	3036(1)	19(1)
N(11)	10255(1)	3311(1)	4665(1)	22(1)
O(11)	4304(1)	3349(1)	5425(1)	27(1)
C(12)	9001(1)	287(2)	3467(1)	24(1)
N(12)	6498(1)	1179(1)	5896(1)	18(1)
O(12)	4546(1)	1524(1)	5970(1)	22(1)
C(14)	9286(1)	4936(1)	3015(1)	18(1)
N(14)	7606(1)	567(1)	6467(1)	24(1)
C(13)	9779(1)	396(2)	3874(1)	27(1)
N(13)	7144(1)	1723(1)	6258(1)	22(1)
C(15)	9920(1)	4203(2)	2732(1)	22(1)
C(18)	10626(1)	6644(2)	3337(1)	23(1)
C(17)	11253(1)	5932(2)	3063(1)	25(1)
C(16)	10904(1)	4726(2)	2765(1)	24(1)
C(19)	9618(1)	6122(1)	3312(1)	19(1)

C(21)	10425(1)	4291(2)	4280(1)	27(1)
C(22)	11101(1)	2403(2)	4875(1)	25(1)
C(23)	9309(1)	3155(1)	4795(1)	19(1)
C(24)	8479(1)	4023(2)	4592(1)	21(1)
C(25)	7550(1)	3927(2)	4754(1)	21(1)
C(26)	7361(1)	2951(1)	5116(1)	19(1)
C(27)	8179(1)	2027(2)	5301(1)	20(1)
C(28)	9115(1)	2119(2)	5145(1)	21(1)
C(29)	6354(1)	2970(1)	5258(1)	20(1)
C(30)	5936(1)	2213(1)	5590(1)	19(1)
C(31)	4852(1)	2447(2)	5648(1)	20(1)
C(32)	3462(1)	1508(2)	6015(1)	26(1)
C(33)	2940(1)	385(2)	5677(1)	34(1)
C(34)	6556(1)	-382(2)	5880(1)	18(1)
C(35)	6074(1)	-1486(2)	5586(1)	22(1)
C(36)	6336(1)	-2991(2)	5684(1)	27(1)
C(37)	7043(1)	-3395(2)	6058(1)	28(1)
C(38)	7519(1)	-2305(2)	6342(1)	26(1)
C(39)	7267(1)	-767(2)	6247(1)	21(1)

Table S6. Bond lengths [Å] and angles [°] for **7a** derivative.

O(1)-C(11)	1.2106(16)
N(1)-C(3)	1.3644(17)
N(1)-C(2)	1.4512(17)
N(1)-C(1)	1.4523(17)
C(1)-H(1A)	0.9600
C(1)-H(1B)	0.9600
C(1)-H(1C)	0.9600
N(2)-C(14)	1.3654(16)
N(2)-N(3)	1.3692(15)
N(2)-C(10)	1.4260(16)
O(2)-C(11)	1.3408(15)
O(2)-C(12)	1.4544(15)
C(2)-H(2A)	0.9600
C(2)-H(2B)	0.9600
C(2)-H(2C)	0.9600
N(3)-N(4)	1.3017(15)
C(3)-C(4)	1.4089(18)
C(3)-C(8)	1.4132(18)
N(4)-C(19)	1.3806(17)
C(4)-C(5)	1.3712(19)
C(4)-H(4)	0.9300
C(5)-C(6)	1.4025(18)
C(5)-H(5)	0.9300
C(6)-C(7)	1.4050(18)
C(6)-C(9)	1.4421(18)
C(7)-C(8)	1.3745(18)
C(7)-H(7)	0.9300
C(8)-H(8)	0.9300
C(9)-C(10)	1.3446(18)
C(9)-H(9)	0.9300
C(10)-C(11)	1.4794(18)
N(11)-C(23)	1.3573(17)
N(11)-C(22)	1.4519(17)
N(11)-C(21)	1.4552(17)

O(11)-C(31)	1.2111(16)
C(12)-C(13)	1.4934(19)
C(12)-H(12A)	0.9700
C(12)-H(12B)	0.9700
N(12)-C(34)	1.3624(17)
N(12)-N(13)	1.3724(15)
N(12)-C(30)	1.4235(16)
O(12)-C(31)	1.3415(16)
O(12)-C(32)	1.4553(16)
C(14)-C(19)	1.3915(18)
C(14)-C(15)	1.4001(18)
N(14)-N(13)	1.2980(16)
N(14)-C(39)	1.3801(18)
C(13)-H(13A)	0.9600
C(13)-H(13B)	0.9600
C(13)-H(13C)	0.9600
C(15)-C(16)	1.3731(19)
C(15)-H(15)	0.9300
C(18)-C(17)	1.370(2)
C(18)-C(19)	1.4035(19)
C(18)-H(18)	0.9300
C(17)-C(16)	1.412(2)
C(17)-H(17)	0.9300
C(16)-H(16)	0.9300
C(21)-H(21A)	0.9600
C(21)-H(21B)	0.9600
C(21)-H(21C)	0.9600
C(22)-H(22A)	0.9600
C(22)-H(22B)	0.9600
C(22)-H(22C)	0.9600
C(23)-C(24)	1.4112(18)
C(23)-C(28)	1.4143(18)
C(24)-C(25)	1.3704(19)
C(24)-H(24)	0.9300
C(25)-C(26)	1.4079(19)
C(25)-H(25)	0.9300

C(26)-C(27)	1.4082(18)
C(26)-C(29)	1.4421(18)
C(27)-C(28)	1.3707(19)
C(27)-H(27)	0.9300
C(28)-H(28)	0.9300
C(29)-C(30)	1.3490(19)
C(29)-H(29)	0.9300
C(30)-C(31)	1.4779(18)
C(32)-C(33)	1.506(2)
C(32)-H(32A)	0.9700
C(32)-H(32B)	0.9700
C(33)-H(33A)	0.9600
C(33)-H(33B)	0.9600
C(33)-H(33C)	0.9600
C(34)-C(39)	1.3920(18)
C(34)-C(35)	1.3985(19)
C(35)-C(36)	1.378(2)
C(35)-H(35)	0.9300
C(36)-C(37)	1.407(2)
C(36)-H(36)	0.9300
C(37)-C(38)	1.370(2)
C(37)-H(37)	0.9300
C(38)-C(39)	1.4012(19)
C(38)-H(38)	0.9300

C(3)-N(1)-C(2)	120.89(11)
C(3)-N(1)-C(1)	120.57(11)
C(2)-N(1)-C(1)	117.79(11)
N(1)-C(1)-H(1A)	109.5
N(1)-C(1)-H(1B)	109.5
H(1A)-C(1)-H(1B)	109.5
N(1)-C(1)-H(1C)	109.5
H(1A)-C(1)-H(1C)	109.5
H(1B)-C(1)-H(1C)	109.5
C(14)-N(2)-N(3)	109.82(10)
C(14)-N(2)-C(10)	127.99(11)

N(3)-N(2)-C(10)	121.96(10)
C(11)-O(2)-C(12)	115.36(10)
N(1)-C(2)-H(2A)	109.5
N(1)-C(2)-H(2B)	109.5
H(2A)-C(2)-H(2B)	109.5
N(1)-C(2)-H(2C)	109.5
H(2A)-C(2)-H(2C)	109.5
H(2B)-C(2)-H(2C)	109.5
N(4)-N(3)-N(2)	108.80(10)
N(1)-C(3)-C(4)	121.48(12)
N(1)-C(3)-C(8)	121.45(12)
C(4)-C(3)-C(8)	117.07(12)
N(3)-N(4)-C(19)	108.32(10)
C(5)-C(4)-C(3)	120.84(12)
C(5)-C(4)-H(4)	119.6
C(3)-C(4)-H(4)	119.6
C(4)-C(5)-C(6)	122.57(12)
C(4)-C(5)-H(5)	118.7
C(6)-C(5)-H(5)	118.7
C(5)-C(6)-C(7)	116.42(12)
C(5)-C(6)-C(9)	117.05(11)
C(7)-C(6)-C(9)	126.52(12)
C(8)-C(7)-C(6)	121.86(12)
C(8)-C(7)-H(7)	119.1
C(6)-C(7)-H(7)	119.1
C(7)-C(8)-C(3)	121.22(12)
C(7)-C(8)-H(8)	119.4
C(3)-C(8)-H(8)	119.4
C(10)-C(9)-C(6)	133.55(12)
C(10)-C(9)-H(9)	113.2
C(6)-C(9)-H(9)	113.2
C(9)-C(10)-N(2)	123.99(11)
C(9)-C(10)-C(11)	119.31(11)
N(2)-C(10)-C(11)	116.69(11)
O(1)-C(11)-O(2)	123.63(12)
O(1)-C(11)-C(10)	124.23(12)

O(2)-C(11)-C(10)	112.14(11)
C(23)-N(11)-C(22)	121.20(11)
C(23)-N(11)-C(21)	120.39(11)
C(22)-N(11)-C(21)	118.16(11)
O(2)-C(12)-C(13)	107.68(11)
O(2)-C(12)-H(12A)	110.2
C(13)-C(12)-H(12A)	110.2
O(2)-C(12)-H(12B)	110.2
C(13)-C(12)-H(12B)	110.2
H(12A)-C(12)-H(12B)	108.5
C(34)-N(12)-N(13)	109.81(10)
C(34)-N(12)-C(30)	129.52(11)
N(13)-N(12)-C(30)	120.55(10)
C(31)-O(12)-C(32)	116.65(10)
N(2)-C(14)-C(19)	104.32(11)
N(2)-C(14)-C(15)	132.69(12)
C(19)-C(14)-C(15)	122.93(12)
N(13)-N(14)-C(39)	108.55(10)
C(12)-C(13)-H(13A)	109.5
C(12)-C(13)-H(13B)	109.5
H(13A)-C(13)-H(13B)	109.5
C(12)-C(13)-H(13C)	109.5
H(13A)-C(13)-H(13C)	109.5
H(13B)-C(13)-H(13C)	109.5
N(14)-N(13)-N(12)	108.68(10)
C(16)-C(15)-C(14)	115.66(12)
C(16)-C(15)-H(15)	122.2
C(14)-C(15)-H(15)	122.2
C(17)-C(18)-C(19)	117.22(12)
C(17)-C(18)-H(18)	121.4
C(19)-C(18)-H(18)	121.4
C(18)-C(17)-C(16)	121.58(13)
C(18)-C(17)-H(17)	119.2
C(16)-C(17)-H(17)	119.2
C(15)-C(16)-C(17)	122.24(13)
C(15)-C(16)-H(16)	118.9

C(17)-C(16)-H(16)	118.9
N(4)-C(19)-C(14)	108.71(11)
N(4)-C(19)-C(18)	130.85(12)
C(14)-C(19)-C(18)	120.36(12)
N(11)-C(21)-H(21A)	109.5
N(11)-C(21)-H(21B)	109.5
H(21A)-C(21)-H(21B)	109.5
N(11)-C(21)-H(21C)	109.5
H(21A)-C(21)-H(21C)	109.5
H(21B)-C(21)-H(21C)	109.5
N(11)-C(22)-H(22A)	109.5
N(11)-C(22)-H(22B)	109.5
H(22A)-C(22)-H(22B)	109.5
N(11)-C(22)-H(22C)	109.5
H(22A)-C(22)-H(22C)	109.5
H(22B)-C(22)-H(22C)	109.5
N(11)-C(23)-C(24)	121.78(12)
N(11)-C(23)-C(28)	121.24(12)
C(24)-C(23)-C(28)	116.98(12)
C(25)-C(24)-C(23)	120.43(12)
C(25)-C(24)-H(24)	119.8
C(23)-C(24)-H(24)	119.8
C(24)-C(25)-C(26)	122.89(12)
C(24)-C(25)-H(25)	118.6
C(26)-C(25)-H(25)	118.6
C(25)-C(26)-C(27)	116.31(12)
C(25)-C(26)-C(29)	117.41(11)
C(27)-C(26)-C(29)	126.27(12)
C(28)-C(27)-C(26)	121.48(12)
C(28)-C(27)-H(27)	119.3
C(26)-C(27)-H(27)	119.3
C(27)-C(28)-C(23)	121.75(12)
C(27)-C(28)-H(28)	119.1
C(23)-C(28)-H(28)	119.1
C(30)-C(29)-C(26)	132.20(12)
C(30)-C(29)-H(29)	113.9

C(26)-C(29)-H(29)	113.9
C(29)-C(30)-N(12)	122.74(12)
C(29)-C(30)-C(31)	119.83(12)
N(12)-C(30)-C(31)	117.43(11)
O(11)-C(31)-O(12)	124.16(12)
O(11)-C(31)-C(30)	123.96(12)
O(12)-C(31)-C(30)	111.87(11)
O(12)-C(32)-C(33)	109.36(11)
O(12)-C(32)-H(32A)	109.8
C(33)-C(32)-H(32A)	109.8
O(12)-C(32)-H(32B)	109.8
C(33)-C(32)-H(32B)	109.8
H(32A)-C(32)-H(32B)	108.3
C(32)-C(33)-H(33A)	109.5
C(32)-C(33)-H(33B)	109.5
H(33A)-C(33)-H(33B)	109.5
C(32)-C(33)-H(33C)	109.5
H(33A)-C(33)-H(33C)	109.5
H(33B)-C(33)-H(33C)	109.5
N(12)-C(34)-C(39)	104.41(11)
N(12)-C(34)-C(35)	133.16(12)
C(39)-C(34)-C(35)	122.43(12)
C(36)-C(35)-C(34)	115.99(13)
C(36)-C(35)-H(35)	122.0
C(34)-C(35)-H(35)	122.0
C(35)-C(36)-C(37)	122.06(14)
C(35)-C(36)-H(36)	119.0
C(37)-C(36)-H(36)	119.0
C(38)-C(37)-C(36)	121.59(13)
C(38)-C(37)-H(37)	119.2
C(36)-C(37)-H(37)	119.2
C(37)-C(38)-C(39)	117.25(13)
C(37)-C(38)-H(38)	121.4
C(39)-C(38)-H(38)	121.4
N(14)-C(39)-C(34)	108.55(11)
N(14)-C(39)-C(38)	130.79(12)

C(34)-C(39)-C(38) 120.66(13)

Symmetry transformations used to generate equivalent atoms:

Table S7. Anisotropic displacement parameters ($Å^2$x 10^3) for **7a** derivative. The anisotropic displacement factor exponent takes the form: $-2\pi^2[h^2 a^{*2}U^{11} + ... + 2 h k a^* b^* U^{12}]$.

	U^{11}	U^{22}	U^{33}	U^{23}	U^{13}	U^{12}
O(1)	22(1)	17(1)	34(1)	-3(1)	-1(1)	-3(1)
N(1)	23(1)	18(1)	24(1)	2(1)	-1(1)	1(1)
C(1)	24(1)	26(1)	24(1)	3(1)	-1(1)	4(1)
N(2)	15(1)	17(1)	19(1)	-1(1)	1(1)	-1(1)
O(2)	22(1)	16(1)	26(1)	2(1)	-4(1)	0(1)
C(2)	28(1)	20(1)	29(1)	3(1)	2(1)	1(1)
N(3)	21(1)	16(1)	21(1)	-1(1)	1(1)	2(1)
C(3)	16(1)	20(1)	21(1)	0(1)	6(1)	2(1)
N(4)	22(1)	16(1)	23(1)	0(1)	-1(1)	-1(1)
C(4)	17(1)	23(1)	21(1)	-3(1)	0(1)	-1(1)
C(5)	18(1)	18(1)	25(1)	-3(1)	3(1)	-2(1)
C(6)	16(1)	19(1)	21(1)	0(1)	4(1)	-1(1)
C(7)	17(1)	23(1)	19(1)	-1(1)	0(1)	-2(1)
C(8)	20(1)	17(1)	23(1)	-2(1)	2(1)	-3(1)
C(9)	17(1)	18(1)	22(1)	-2(1)	5(1)	-2(1)
C(10)	17(1)	17(1)	21(1)	-2(1)	4(1)	-2(1)
C(11)	16(1)	20(1)	21(1)	-2(1)	4(1)	1(1)
N(11)	21(1)	21(1)	24(1)	4(1)	5(1)	2(1)
O(11)	21(1)	28(1)	32(1)	3(1)	3(1)	7(1)
C(12)	32(1)	15(1)	25(1)	2(1)	0(1)	4(1)
N(12)	16(1)	19(1)	20(1)	-2(1)	0(1)	-1(1)
O(12)	16(1)	25(1)	26(1)	0(1)	3(1)	1(1)
C(14)	17(1)	16(1)	19(1)	4(1)	0(1)	-1(1)
N(14)	18(1)	30(1)	22(1)	2(1)	1(1)	-1(1)
C(13)	31(1)	22(1)	27(1)	1(1)	-2(1)	4(1)
N(13)	18(1)	27(1)	22(1)	-2(1)	0(1)	-3(1)

C(15)	22(1)	22(1)	21(1)	0(1)	1(1)	-1(1)
C(18)	24(1)	19(1)	26(1)	2(1)	-3(1)	-5(1)
C(17)	17(1)	28(1)	28(1)	8(1)	-1(1)	-4(1)
C(16)	20(1)	30(1)	23(1)	4(1)	4(1)	0(1)
C(19)	20(1)	16(1)	20(1)	3(1)	-1(1)	-1(1)
C(21)	28(1)	25(1)	29(1)	5(1)	10(1)	2(1)
C(22)	21(1)	23(1)	32(1)	3(1)	4(1)	2(1)
C(23)	21(1)	17(1)	19(1)	-4(1)	2(1)	0(1)
C(24)	25(1)	19(1)	20(1)	2(1)	2(1)	1(1)
C(25)	21(1)	19(1)	22(1)	-1(1)	-2(1)	3(1)
C(26)	21(1)	16(1)	20(1)	-2(1)	0(1)	1(1)
C(27)	22(1)	18(1)	21(1)	2(1)	2(1)	1(1)
C(28)	21(1)	18(1)	22(1)	1(1)	0(1)	3(1)
C(29)	20(1)	16(1)	23(1)	-3(1)	-2(1)	2(1)
C(30)	18(1)	16(1)	23(1)	-4(1)	0(1)	1(1)
C(31)	21(1)	18(1)	21(1)	-5(1)	2(1)	0(1)
C(32)	17(1)	29(1)	32(1)	-1(1)	6(1)	1(1)
C(33)	24(1)	40(1)	39(1)	1(1)	-1(1)	-7(1)
C(34)	15(1)	19(1)	21(1)	1(1)	6(1)	1(1)
C(35)	21(1)	23(1)	24(1)	-2(1)	3(1)	0(1)
C(36)	28(1)	22(1)	33(1)	-4(1)	10(1)	-1(1)
C(37)	28(1)	22(1)	36(1)	7(1)	14(1)	5(1)
C(38)	20(1)	31(1)	28(1)	10(1)	8(1)	6(1)
C(39)	15(1)	27(1)	22(1)	3(1)	6(1)	0(1)

Table S8. Hydrogen coordinates (x 10^4) and isotropic displacement parameters (Å^{2}x 10^3) for **7a** derivative.

	x	y	z	U(eq)
H(1A)	3962	9819	1199	37
H(1B)	3654	8238	1397	37
H(1C)	4514	8311	1073	37
H(2A)	5980	10725	1696	38
H(2B)	5389	10761	2129	38
H(2C)	4857	11323	1655	38
H(4)	4575	6135	1583	24
H(5)	5328	4088	1964	24
H(7)	7219	6904	2727	24
H(8)	6446	8977	2363	24
H(9)	6432	2971	2504	23
H(12A)	9288	-213	3216	29
H(12B)	8423	-310	3541	29
H(13A)	10004	-617	3966	40
H(13B)	9487	887	4120	40
H(13C)	10348	986	3796	40
H(15)	9689	3412	2535	26
H(18)	10858	7440	3532	28
H(17)	11925	6252	3072	30
H(16)	11355	4270	2586	29
H(21A)	10097	3850	4003	40
H(21B)	11141	4376	4260	40
H(21C)	10147	5291	4324	40
H(22A)	10985	1339	4801	38
H(22B)	11161	2534	5201	38
H(22C)	11718	2735	4762	38
H(24)	8562	4665	4346	25
H(25)	7022	4534	4619	25
H(27)	8082	1339	5534	24
H(28)	9635	1483	5273	25
H(29)	5907	3629	5087	24

H(32A)	3180	2527	5957	31
H(32B)	3352	1212	6323	31
H(33A)	3030	705	5372	52
H(33B)	2228	352	5712	52
H(33C)	3230	-617	5732	52
H(35)	5605	-1221	5340	27
H(36)	6035	-3765	5497	33
H(37)	7191	-4427	6114	34
H(38)	7991	-2575	6587	31

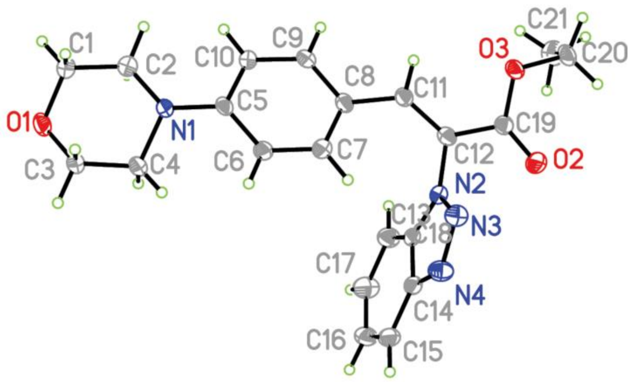

Fig S21. Single-crystal X-ray analysis of **7d** derivative (CCDC 1882467)

Table S9. Atomic coordinates (x 10^4) and equivalent isotropic displacement parameters ($Å^2$x 10^3) for **7d** derivative. U(eq) is defined as one third of the trace of the orthogonalized U^{ij} tensor.

	x	y	z	U(eq)
O(1)	6630(1)	3687(3)	4694(1)	27(1)
N(1)	8199(1)	2770(4)	4565(1)	20(1)
C(1)	7197(2)	4574(5)	4942(1)	27(1)
O(2)	12526(1)	988(3)	3160(1)	30(1)
N(2)	11113(1)	-197(4)	3418(1)	18(1)
C(2)	7949(2)	4803(5)	4728(1)	27(1)
C(3)	6863(2)	1628(5)	4579(1)	28(1)
O(3)	12719(1)	3008(3)	3702(1)	24(1)
N(3)	11162(1)	-2267(4)	3532(1)	23(1)
C(4)	7593(2)	1691(5)	4344(1)	28(1)
N(4)	10707(1)	-3390(4)	3312(1)	26(1)
C(5)	8930(1)	2706(4)	4400(1)	19(1)
C(6)	9178(2)	959(5)	4179(1)	21(1)
C(7)	9902(2)	867(5)	4017(1)	22(1)
C(8)	10417(2)	2516(5)	4065(1)	20(1)
C(9)	10181(2)	4228(5)	4293(1)	24(1)
C(10)	9456(2)	4346(5)	4457(1)	23(1)
C(11)	11189(2)	2617(4)	3900(1)	19(1)
O(11)	5750(1)	1086(4)	5341(1)	28(1)
N(11)	4194(1)	2174(4)	5468(1)	22(1)
C(12)	11518(1)	1414(4)	3621(1)	20(1)
O(12)	-113(1)	4629(4)	6857(1)	34(1)
N(12)	1314(1)	5631(4)	6603(1)	19(1)
C(14)	10354(2)	-2048(4)	3045(1)	19(1)
N(14)	1531(2)	8863(4)	6788(1)	28(1)
C(13)	10612(1)	1(4)	3110(1)	17(1)
O(13)	-299(1)	2362(4)	6348(1)	34(1)
N(13)	1127(1)	7714(4)	6547(1)	25(1)
C(16)	9570(2)	-778(5)	2529(1)	29(1)
C(15)	9822(2)	-2468(5)	2745(1)	25(1)
C(18)	10366(2)	1742(5)	2890(1)	27(1)

C(17)	9840(2)	1293(5)	2599(1)	32(1)
C(19)	12300(2)	1766(4)	3466(1)	21(1)
C(20)	13477(2)	3578(5)	3557(1)	26(1)
C(21)	13440(2)	5552(5)	3309(1)	35(1)
C(31)	5171(2)	249(5)	5094(1)	30(1)
C(32)	4421(2)	96(5)	5310(1)	28(1)
C(33)	5545(2)	3165(5)	5454(1)	30(1)
C(34)	4815(2)	3182(5)	5692(1)	29(1)
C(35)	3464(2)	2319(4)	5634(1)	21(1)
C(36)	3234(2)	4163(5)	5838(1)	22(1)
C(37)	2518(2)	4340(4)	6004(1)	21(1)
C(38)	1984(2)	2686(4)	5975(1)	19(1)
C(39)	2206(2)	893(5)	5763(1)	24(1)
C(40)	2924(2)	699(5)	5596(1)	24(1)
C(41)	1219(2)	2691(4)	6145(1)	20(1)
C(42)	900(2)	3997(4)	6411(1)	20(1)
C(43)	1848(2)	5457(4)	6896(1)	20(1)
C(44)	1985(2)	7524(4)	7014(1)	22(1)
C(45)	2506(2)	7974(5)	7319(1)	30(1)
C(46)	2888(2)	6294(6)	7477(1)	32(1)
C(47)	2759(2)	4203(5)	7348(1)	32(1)
C(48)	2231(2)	3735(5)	7058(1)	26(1)
C(49)	114(2)	3713(5)	6569(1)	24(1)
C(50)	-1048(2)	1747(6)	6494(1)	42(1)
C(51)	-1009(2)	-225(6)	6718(1)	51(1)

Table S10. Bond lengths [Å] and angles [°] for **7d** derivative.

O(1)-C(1)	1.416(4)
O(1)-C(3)	1.419(4)
N(1)-C(5)	1.400(3)
N(1)-C(2)	1.465(4)
N(1)-C(4)	1.468(3)
C(1)-C(2)	1.513(4)
C(1)-H(1A)	0.9900
C(1)-H(1AB)	0.9900
O(2)-C(19)	1.209(3)
N(2)-C(13)	1.365(3)
N(2)-N(3)	1.368(3)
N(2)-C(12)	1.420(3)
C(2)-H(2A)	0.9900
C(2)-H(2AB)	0.9900
C(3)-C(4)	1.508(4)
C(3)-H(3A)	0.9900
C(3)-H(3AB)	0.9900
O(3)-C(19)	1.339(3)
O(3)-C(20)	1.463(3)
N(3)-N(4)	1.302(3)
C(4)-H(4A)	0.9900
C(4)-H(4AB)	0.9900
N(4)-C(14)	1.383(4)
C(5)-C(10)	1.401(4)
C(5)-C(6)	1.403(4)
C(6)-C(7)	1.384(4)
C(6)-H(6)	0.9500
C(7)-C(8)	1.390(4)
C(7)-H(7)	0.9500
C(8)-C(9)	1.392(4)
C(8)-C(11)	1.466(4)
C(9)-C(10)	1.389(4)
C(9)-H(9)	0.9500
C(10)-H(10)	0.9500

C(11)-C(12)	1.342(4)
C(11)-H(11)	0.9500
O(11)-C(33)	1.415(4)
O(11)-C(31)	1.416(4)
N(11)-C(35)	1.402(3)
N(11)-C(34)	1.472(3)
N(11)-C(32)	1.474(4)
C(12)-C(19)	1.486(4)
O(12)-C(49)	1.200(4)
N(12)-C(43)	1.366(4)
N(12)-N(13)	1.371(3)
N(12)-C(42)	1.419(4)
C(14)-C(13)	1.390(4)
C(14)-C(15)	1.402(4)
N(14)-N(13)	1.301(4)
N(14)-C(44)	1.392(4)
C(13)-C(18)	1.397(4)
O(13)-C(49)	1.346(4)
O(13)-C(50)	1.459(4)
C(16)-C(15)	1.366(4)
C(16)-C(17)	1.413(5)
C(16)-H(16)	0.9500
C(15)-H(15)	0.9500
C(18)-C(17)	1.377(4)
C(18)-H(18)	0.9500
C(17)-H(17)	0.9500
C(20)-C(21)	1.505(5)
C(20)-H(20A)	0.9900
C(20)-H(20B)	0.9900
C(21)-H(21A)	0.9800
C(21)-H(21B)	0.9800
C(21)-H(21C)	0.9800
C(31)-C(32)	1.508(4)
C(31)-H(31A)	0.9900
C(31)-H(31B)	0.9900
C(32)-H(32A)	0.9900

C(32)-H(32B)	0.9900
C(33)-C(34)	1.514(4)
C(33)-H(33A)	0.9900
C(33)-H(33B)	0.9900
C(34)-H(34A)	0.9900
C(34)-H(34B)	0.9900
C(35)-C(40)	1.401(4)
C(35)-C(36)	1.412(4)
C(36)-C(37)	1.382(4)
C(36)-H(36)	0.9500
C(37)-C(38)	1.408(4)
C(37)-H(37)	0.9500
C(38)-C(39)	1.395(4)
C(38)-C(41)	1.460(4)
C(39)-C(40)	1.386(4)
C(39)-H(39)	0.9500
C(40)-H(40)	0.9500
C(41)-C(42)	1.342(4)
C(41)-H(41)	0.9500
C(42)-C(49)	1.490(4)
C(43)-C(44)	1.388(4)
C(43)-C(48)	1.392(4)
C(44)-C(45)	1.405(4)
C(45)-C(46)	1.364(5)
C(45)-H(45)	0.9500
C(46)-C(47)	1.410(5)
C(46)-H(46)	0.9500
C(47)-C(48)	1.380(4)
C(47)-H(47)	0.9500
C(48)-H(48)	0.9500
C(50)-C(51)	1.458(5)
C(50)-H(50A)	0.9900
C(50)-H(50B)	0.9900
C(51)-H(51A)	0.9800
C(51)-H(51B)	0.9800
C(51)-H(51C)	0.9800

C(1)-O(1)-C(3)	108.8(2)
C(5)-N(1)-C(2)	116.7(2)
C(5)-N(1)-C(4)	116.7(2)
C(2)-N(1)-C(4)	112.4(2)
O(1)-C(1)-C(2)	111.7(2)
O(1)-C(1)-H(1A)	109.3
C(2)-C(1)-H(1A)	109.3
O(1)-C(1)-H(1AB)	109.3
C(2)-C(1)-H(1AB)	109.3
H(1A)-C(1)-H(1AB)	107.9
C(13)-N(2)-N(3)	110.1(2)
C(13)-N(2)-C(12)	128.6(2)
N(3)-N(2)-C(12)	121.3(2)
N(1)-C(2)-C(1)	110.9(3)
N(1)-C(2)-H(2A)	109.5
C(1)-C(2)-H(2A)	109.5
N(1)-C(2)-H(2AB)	109.5
C(1)-C(2)-H(2AB)	109.5
H(2A)-C(2)-H(2AB)	108.1
O(1)-C(3)-C(4)	111.4(3)
O(1)-C(3)-H(3A)	109.3
C(4)-C(3)-H(3A)	109.3
O(1)-C(3)-H(3AB)	109.3
C(4)-C(3)-H(3AB)	109.3
H(3A)-C(3)-H(3AB)	108.0
C(19)-O(3)-C(20)	116.4(2)
N(4)-N(3)-N(2)	108.8(2)
N(1)-C(4)-C(3)	111.1(2)
N(1)-C(4)-H(4A)	109.4
C(3)-C(4)-H(4A)	109.4
N(1)-C(4)-H(4AB)	109.4
C(3)-C(4)-H(4AB)	109.4
H(4A)-C(4)-H(4AB)	108.0
N(3)-N(4)-C(14)	108.2(2)
N(1)-C(5)-C(10)	121.9(2)
N(1)-C(5)-C(6)	121.2(2)

C(10)-C(5)-C(6)	116.8(2)
C(7)-C(6)-C(5)	121.8(3)
C(7)-C(6)-H(6)	119.1
C(5)-C(6)-H(6)	119.1
C(6)-C(7)-C(8)	121.4(3)
C(6)-C(7)-H(7)	119.3
C(8)-C(7)-H(7)	119.3
C(7)-C(8)-C(9)	117.0(2)
C(7)-C(8)-C(11)	126.2(3)
C(9)-C(8)-C(11)	116.8(2)
C(10)-C(9)-C(8)	122.3(3)
C(10)-C(9)-H(9)	118.9
C(8)-C(9)-H(9)	118.9
C(9)-C(10)-C(5)	120.7(3)
C(9)-C(10)-H(10)	119.7
C(5)-C(10)-H(10)	119.7
C(12)-C(11)-C(8)	129.7(3)
C(12)-C(11)-H(11)	115.1
C(8)-C(11)-H(11)	115.1
C(33)-O(11)-C(31)	108.9(2)
C(35)-N(11)-C(34)	116.3(2)
C(35)-N(11)-C(32)	116.8(2)
C(34)-N(11)-C(32)	111.8(2)
C(11)-C(12)-N(2)	122.1(2)
C(11)-C(12)-C(19)	124.1(2)
N(2)-C(12)-C(19)	113.6(2)
C(43)-N(12)-N(13)	110.0(2)
C(43)-N(12)-C(42)	128.5(2)
N(13)-N(12)-C(42)	120.9(2)
N(4)-C(14)-C(13)	108.8(2)
N(4)-C(14)-C(15)	130.6(3)
C(13)-C(14)-C(15)	120.5(3)
N(13)-N(14)-C(44)	108.4(2)
N(2)-C(13)-C(14)	104.2(2)
N(2)-C(13)-C(18)	132.5(3)
C(14)-C(13)-C(18)	123.3(2)

C(49)-O(13)-C(50)	117.8(3)
N(14)-N(13)-N(12)	108.7(2)
C(15)-C(16)-C(17)	121.9(3)
C(15)-C(16)-H(16)	119.1
C(17)-C(16)-H(16)	119.1
C(16)-C(15)-C(14)	116.8(3)
C(16)-C(15)-H(15)	121.6
C(14)-C(15)-H(15)	121.6
C(17)-C(18)-C(13)	115.1(3)
C(17)-C(18)-H(18)	122.4
C(13)-C(18)-H(18)	122.4
C(18)-C(17)-C(16)	122.3(3)
C(18)-C(17)-H(17)	118.8
C(16)-C(17)-H(17)	118.8
O(2)-C(19)-O(3)	124.5(2)
O(2)-C(19)-C(12)	122.8(3)
O(3)-C(19)-C(12)	112.7(2)
O(3)-C(20)-C(21)	110.7(2)
O(3)-C(20)-H(20A)	109.5
C(21)-C(20)-H(20A)	109.5
O(3)-C(20)-H(20B)	109.5
C(21)-C(20)-H(20B)	109.5
H(20A)-C(20)-H(20B)	108.1
C(20)-C(21)-H(21A)	109.5
C(20)-C(21)-H(21B)	109.5
H(21A)-C(21)-H(21B)	109.5
C(20)-C(21)-H(21C)	109.5
H(21A)-C(21)-H(21C)	109.5
H(21B)-C(21)-H(21C)	109.5
O(11)-C(31)-C(32)	111.5(2)
O(11)-C(31)-H(31A)	109.3
C(32)-C(31)-H(31A)	109.3
O(11)-C(31)-H(31B)	109.3
C(32)-C(31)-H(31B)	109.3
H(31A)-C(31)-H(31B)	108.0
N(11)-C(32)-C(31)	110.7(3)

N(11)-C(32)-H(32A)	109.5
C(31)-C(32)-H(32A)	109.5
N(11)-C(32)-H(32B)	109.5
C(31)-C(32)-H(32B)	109.5
H(32A)-C(32)-H(32B)	108.1
O(11)-C(33)-C(34)	111.3(3)
O(11)-C(33)-H(33A)	109.4
C(34)-C(33)-H(33A)	109.4
O(11)-C(33)-H(33B)	109.4
C(34)-C(33)-H(33B)	109.4
H(33A)-C(33)-H(33B)	108.0
N(11)-C(34)-C(33)	110.6(2)
N(11)-C(34)-H(34A)	109.5
C(33)-C(34)-H(34A)	109.5
N(11)-C(34)-H(34B)	109.5
C(33)-C(34)-H(34B)	109.5
H(34A)-C(34)-H(34B)	108.1
C(40)-C(35)-N(11)	122.3(2)
C(40)-C(35)-C(36)	117.1(2)
N(11)-C(35)-C(36)	120.6(2)
C(37)-C(36)-C(35)	121.5(3)
C(37)-C(36)-H(36)	119.2
C(35)-C(36)-H(36)	119.2
C(36)-C(37)-C(38)	121.2(3)
C(36)-C(37)-H(37)	119.4
C(38)-C(37)-H(37)	119.4
C(39)-C(38)-C(37)	117.0(2)
C(39)-C(38)-C(41)	117.4(3)
C(37)-C(38)-C(41)	125.7(2)
C(40)-C(39)-C(38)	122.2(3)
C(40)-C(39)-H(39)	118.9
C(38)-C(39)-H(39)	118.9
C(39)-C(40)-C(35)	120.9(3)
C(39)-C(40)-H(40)	119.5
C(35)-C(40)-H(40)	119.5
C(42)-C(41)-C(38)	130.4(3)

C(42)-C(41)-H(41)	114.8
C(38)-C(41)-H(41)	114.8
C(41)-C(42)-N(12)	122.6(2)
C(41)-C(42)-C(49)	123.5(3)
N(12)-C(42)-C(49)	113.6(2)
N(12)-C(43)-C(44)	104.6(2)
N(12)-C(43)-C(48)	132.7(3)
C(44)-C(43)-C(48)	122.7(3)
C(43)-C(44)-N(14)	108.4(2)
C(43)-C(44)-C(45)	121.0(3)
N(14)-C(44)-C(45)	130.6(3)
C(46)-C(45)-C(44)	116.6(3)
C(46)-C(45)-H(45)	121.7
C(44)-C(45)-H(45)	121.7
C(45)-C(46)-C(47)	122.1(3)
C(45)-C(46)-H(46)	119.0
C(47)-C(46)-H(46)	119.0
C(48)-C(47)-C(46)	121.8(3)
C(48)-C(47)-H(47)	119.1
C(46)-C(47)-H(47)	119.1
C(47)-C(48)-C(43)	115.8(3)
C(47)-C(48)-H(48)	122.1
C(43)-C(48)-H(48)	122.1
O(12)-C(49)-O(13)	125.3(3)
O(12)-C(49)-C(42)	122.6(3)
O(13)-C(49)-C(42)	112.1(2)
C(51)-C(50)-O(13)	111.2(3)
C(51)-C(50)-H(50A)	109.4
O(13)-C(50)-H(50A)	109.4
C(51)-C(50)-H(50B)	109.4
O(13)-C(50)-H(50B)	109.4
H(50A)-C(50)-H(50B)	108.0
C(50)-C(51)-H(51A)	109.5
C(50)-C(51)-H(51B)	109.5
H(51A)-C(51)-H(51B)	109.5
C(50)-C(51)-H(51C)	109.5

H(51A)-C(51)-H(51C)	109.5
H(51B)-C(51)-H(51C)	109.5

Symmetry transformations used to generate equivalent atoms:

Table S11. Anisotropic displacement parameters ($Å^2$x 10^3) for **7d** derivative. The anisotropic displacement factor exponent takes the form: $-2\pi^2$[h^2 $a^{*2}U^{11}$ + ... + 2 h k a* b* U^{12}].

	U^{11}	U^{22}	U^{33}	U^{23}	U^{13}	U^{12}
O(1)	15(1)	41(1)	26(1)	-1(1)	-3(1)	4(1)
N(1)	15(1)	26(1)	19(1)	-3(1)	-1(1)	0(1)
C(1)	20(1)	34(2)	28(2)	-7(1)	2(1)	3(1)
O(2)	24(1)	39(1)	26(1)	-9(1)	7(1)	-4(1)
N(2)	18(1)	18(1)	18(1)	0(1)	1(1)	1(1)
C(2)	22(1)	31(2)	28(1)	-7(1)	1(1)	1(1)
C(3)	17(1)	41(2)	27(1)	-9(1)	2(1)	-1(1)
O(3)	16(1)	35(1)	22(1)	-4(1)	2(1)	-6(1)
N(3)	28(1)	19(1)	24(1)	3(1)	-2(1)	0(1)
C(4)	16(1)	40(2)	27(1)	-10(1)	1(1)	-4(1)
N(4)	32(1)	21(1)	25(1)	1(1)	-4(1)	-3(1)
C(5)	14(1)	27(2)	14(1)	1(1)	-3(1)	1(1)
C(6)	19(1)	24(2)	21(1)	-2(1)	2(1)	-4(1)
C(7)	22(1)	25(2)	21(1)	-3(1)	2(1)	0(1)
C(8)	16(1)	25(2)	18(1)	1(1)	-1(1)	0(1)
C(9)	17(1)	28(2)	26(2)	-6(1)	-1(1)	-4(1)
C(10)	18(1)	27(2)	25(1)	-7(1)	0(1)	1(1)
C(11)	17(1)	22(2)	19(1)	0(1)	-1(1)	0(1)
O(11)	16(1)	41(1)	28(1)	-3(1)	0(1)	6(1)
N(11)	15(1)	29(1)	22(1)	-4(1)	1(1)	0(1)
C(12)	18(1)	23(2)	18(1)	1(1)	-2(1)	-2(1)
O(12)	27(1)	47(1)	27(1)	2(1)	8(1)	6(1)
N(12)	18(1)	20(1)	19(1)	1(1)	1(1)	3(1)
C(14)	19(1)	20(1)	19(1)	-2(1)	2(1)	0(1)
N(14)	38(1)	22(1)	24(1)	0(1)	-1(1)	0(1)

C(13)	17(1)	20(1)	15(1)	-2(1)	2(1)	1(1)
O(13)	17(1)	30(1)	53(1)	-2(1)	8(1)	-5(1)
N(13)	31(1)	20(1)	25(1)	1(1)	0(1)	4(1)
C(16)	28(2)	31(2)	28(1)	-6(1)	-9(1)	2(1)
C(15)	27(1)	23(2)	24(1)	-5(1)	0(1)	-3(1)
C(18)	29(2)	21(2)	30(1)	-2(1)	-5(1)	1(1)
C(17)	41(2)	24(2)	32(2)	1(1)	-12(1)	6(1)
C(19)	20(1)	24(1)	18(1)	0(1)	-1(1)	2(1)
C(20)	14(1)	38(2)	26(1)	-2(1)	3(1)	-5(1)
C(21)	33(2)	40(2)	32(2)	2(1)	7(1)	-6(2)
C(31)	22(2)	39(2)	28(2)	-8(1)	2(1)	4(1)
C(32)	21(2)	32(2)	32(2)	-9(1)	5(1)	0(1)
C(33)	18(1)	41(2)	31(2)	-8(1)	1(1)	-2(1)
C(34)	17(1)	44(2)	27(1)	-11(1)	2(1)	-2(1)
C(35)	17(1)	27(2)	19(1)	-1(1)	-1(1)	1(1)
C(36)	18(1)	24(2)	23(1)	0(1)	2(1)	-3(1)
C(37)	20(1)	21(1)	20(1)	-2(1)	2(1)	0(1)
C(38)	15(1)	25(2)	18(1)	0(1)	-2(1)	1(1)
C(39)	17(1)	27(2)	27(2)	-6(1)	-3(1)	-3(1)
C(40)	18(1)	26(2)	28(1)	-8(1)	-2(1)	-1(1)
C(41)	17(1)	21(2)	21(1)	3(1)	-4(1)	1(1)
C(42)	16(1)	21(2)	22(1)	4(1)	-1(1)	1(1)
C(43)	18(1)	22(1)	20(1)	1(1)	4(1)	0(1)
C(44)	24(1)	22(1)	20(1)	-1(1)	5(1)	-2(1)
C(45)	38(2)	31(2)	22(2)	-1(1)	0(1)	-10(1)
C(46)	36(2)	37(2)	23(1)	1(1)	-8(1)	-9(1)
C(47)	32(2)	31(2)	33(2)	3(1)	-12(1)	-1(1)
C(48)	22(1)	24(2)	32(2)	0(1)	-4(1)	2(1)
C(49)	19(1)	25(2)	28(2)	9(1)	3(1)	3(1)
C(50)	21(2)	38(2)	68(2)	8(2)	12(2)	-5(2)
C(51)	40(2)	42(2)	70(3)	12(2)	26(2)	1(2)

Table S12. Hydrogen coordinates (x10^4) and isotropic displacement parameters (Å^{2}x 10^3) for **7d** derivatives.

	x	y	z	U(eq)
H(1A)	7267	3659	5177	33
H(1AB)	7026	5980	5035	33
H(2A)	7895	5842	4510	32
H(2AB)	8339	5343	4914	32
H(3A)	6458	966	4417	34
H(3AB)	6938	748	4819	34
H(4A)	7756	229	4281	33
H(4AB)	7503	2440	4090	33
H(6)	8839	-193	4138	26
H(7)	10049	-348	3870	27
H(9)	10528	5355	4339	28
H(10)	9316	5550	4608	28
H(11)	11504	3697	4008	23
H(16)	9203	-1003	2327	34
H(15)	9645	-3861	2695	30
H(18)	10549	3134	2937	32
H(17)	9652	2416	2439	39
H(20A)	13685	2402	3396	31
H(20B)	13823	3811	3784	31
H(21A)	13958	6070	3259	53
H(21B)	13149	6638	3450	53
H(21C)	13191	5235	3056	53
H(31A)	5324	-1175	5001	35
H(31B)	5110	1166	4858	35
H(32A)	4024	-426	5126	34
H(32B)	4467	-929	5530	34
H(33A)	5479	4045	5213	36
H(33B)	5961	3791	5614	36
H(34A)	4894	2415	5945	35
H(34B)	4674	4659	5756	35
H(36)	3581	5310	5861	26

H(37)	2383	5600	6140	25
H(39)	1852	-232	5732	29
H(40)	3052	-549	5455	29
H(41)	896	1592	6053	24
H(45)	2588	9376	7411	36
H(46)	3252	6540	7680	38
H(47)	3044	3086	7463	39
H(48)	2135	2326	6974	32
H(50A)	-1251	2880	6667	51
H(50B)	-1401	1572	6268	51
H(51A)	-1526	-744	6768	76
H(51B)	-750	25	6970	76
H(51C)	-726	-1280	6564	76

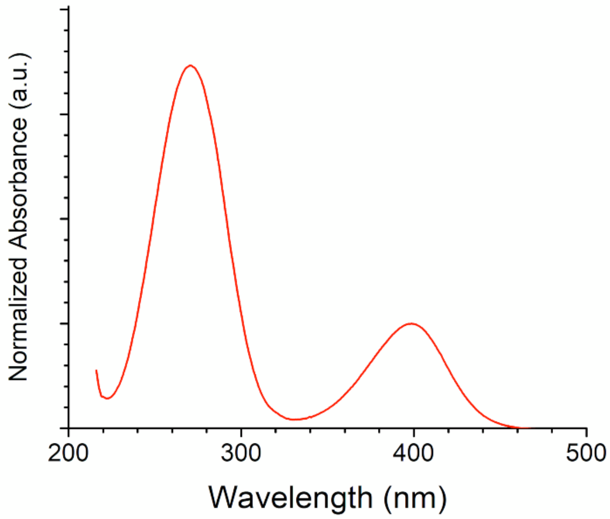

Fig S20. Absorption spectra of **6b** derivative

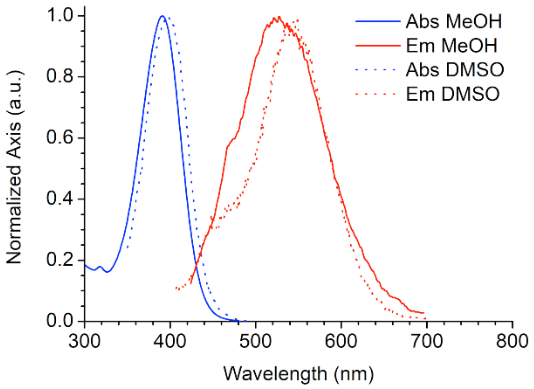

Fig S21. Absorption and emission spectra of **6a** derivative in (solid line) methanol and (dot line) DMSO solution
